# Reducing chronic disease through changes in food aid: A microsimulation of nutrition and cardiometabolic disease among Palestinian refugees in the Middle East

**DOI:** 10.1371/journal.pmed.1002700

**Published:** 2018-11-20

**Authors:** Sanjay Basu, John S. Yudkin, Seth A. Berkowitz, Mohammed Jawad, Christopher Millett

**Affiliations:** 1 Stanford University, Stanford, California, United States of America; 2 Harvard Medical School, Boston, Massachusetts, United States of America; 3 University College London, London, United Kingdom; 4 University of North Carolina at Chapel Hill, Chapel Hill, North Carolina, United States of America; 5 Public Health Policy Evaluation Unit, School of Public Health, Imperial College London, London, United Kingdom; 6 Center for Epidemiological Studies in Health and Nutrition, University of São Paulo, São Paulo, Brazil; University of Oxford, UNITED KINGDOM

## Abstract

**Background:**

Type 2 diabetes mellitus and cardiovascular disease and have become leading causes of morbidity and mortality among Palestinian refugees in the Middle East, many of whom live in long-term settlements and receive grain-based food aid. The objective of this study was to estimate changes in type 2 diabetes and cardiovascular disease morbidity and mortality attributable to a transition from traditional food aid to either (i) a debit card restricted to food purchases, (ii) cash, or (iii) an alternative food parcel with less grain and more fruits and vegetables, each valued at $30/person/month.

**Methods and findings:**

An individual-level microsimulation was created to estimate relationships between food aid delivery method, food consumption, type 2 diabetes, and cardiovascular disease morbidity and mortality using demographic data from the United Nations (UN; 2017) on 5,340,443 registered Palestinian refugees in Syria, Jordan, Lebanon, Gaza, and the West Bank, food consumption data (2011–2017) from households receiving traditional food parcel delivery of food aid (*n* = 1,507 households) and electronic debit card delivery of food aid (*n* = 1,047 households), and health data from a random 10% sample of refugees receiving medical care through the UN (2012–2015; *n* = 516,386). Outcome metrics included incidence per 1,000 person-years of hypertension, type 2 diabetes, atherosclerotic cardiovascular disease events, microvascular events (end-stage renal disease, diabetic neuropathy, and proliferative diabetic retinopathy), and all-cause mortality. The model estimated changes in total calories, sodium and potassium intake, fatty acid intake, and overall dietary quality (Mediterranean Dietary Score [MDS]) as mediators to each outcome metric. We did not observe that a change from food parcel to electronic debit card delivery of food aid or to cash aid led to a meaningful change in consumption, biomarkers, or disease outcomes. By contrast, a shift to an alternative food parcel with less grain and more fruits and vegetables was estimated to produce a 0.08 per 1,000 person-years decrease in the incidence of hypertension (95% confidence interval [CI] 0.05–0.11), 0.18 per 1,000 person-years decrease in the incidence of type 2 diabetes (95% CI 0.14–0.22), 0.18 per 1,000 person-years decrease in the incidence of atherosclerotic cardiovascular disease events (95% CI 0.17–0.19), and 0.02 decrease per 1,000 person-years all-cause mortality (95% CI 0.01 decrease to 0.04 increase) among those receiving aid. The benefits of this shift, however, could be neutralized by a small (2%) increase in compensatory (out-of-pocket) increases in consumption of refined grains, fats and oils, or confectionaries. A larger alternative parcel requiring an increase in total food aid expenditure by 27% would be more likely to have a clinically meaningful improvement on type 2 diabetes and cardiovascular disease incidence.

**Conclusions:**

Contrary to the supposition in the literature, our findings do not robustly support the theory that transitioning from traditional food aid to either debit card or cash delivery alone would necessarily reduce chronic disease outcomes. Rather, an alternative food parcel would be more effective, even after matching current budget ceilings. But compensatory increases in consumption of less healthy foods may neutralize the improvements from an alternative food parcel unless total aid funding were increased substantially. Our analysis is limited by uncertainty in estimates of modeling long-term outcomes from shorter-term trials, focusing on diabetes and cardiovascular outcomes for which validated equations are available instead of all nutrition-associated health outcomes, and using data from food frequency questionnaires in the absence of 24-hour dietary recall data.

## Abstract 2

Using a modeling approach, Sanjay Basu and colleagues predict the changes in food aid needed to reduce type 2 diabetes and CVD in Palestinian refugees in the Middle East.

## Author summary

**Why was this study done?:**

- Type 2 diabetes mellitus and cardiovascular disease have become leading causes of morbidity and mortality among Palestinian refugees.
- Traditional food aid is often primarily composed of grain, flour, and rice, which may increase the risk of such chronic diseases.
- It remains unclear how much changes in food aid in delivery method and composition may alter chronic disease risk.

**What did the researchers do and find?:**

- We constructed a microsimulation model to estimate the impact of food aid delivery methods on food consumption, type 2 diabetes, and cardiovascular disease outcomes among the 20- to 79-year old subpopulation receiving food aid (43%) among 5.3 million registered Palestinian refugees in Syria, Jordan, Lebanon, Gaza, and the West Bank.
- The simulation took into account the proportion who received food aid and the dietary patterns among them, including the portion of food acquired and consumed from other sources besides food aid.
- Using the simulation, we did not observe that a change from food parcel to electronic debit card delivery of food aid or to cash aid led to a meaningful change in consumption, biomarkers, or disease outcomes. We did observe that a shift an alternative food parcel with less grain and more fruits and vegetables may avert hypertension and type 2 diabetes but be neutralized through minimal compensatory (out-of-pocket) increases in consumption of refined grains, fats and oils, or confectionaries.
- A larger alternative parcel requiring an increase in total food aid expenditure by 27% would be more likely to have a clinically meaningful improvement on type 2 diabetes and cardiovascular disease.

**What do these findings mean?:**

- An alternative food parcel would be more effective than transitioning from traditional food aid to either debit card or cash delivery from the perspective of reducing chronic disease.
- But compensatory increases in consumption of less healthy foods may neutralize the improvements from an alternative food parcel unless total aid funding were increased substantially.

## Introduction

Increasingly, refugee camps worldwide have become semipermanent or permanent, accompanied by an epidemiologic transition with fewer traumatic injuries, infectious diseases, or malnutrition as well as a much higher rate of chronic disease [1,2]. Type 2 diabetes mellitus and cardiovascular disease have become leading causes of morbidity and mortality among refugees in the Middle East [3–9], and are particularly prevalent among the >5 million registered Palestine refugees who live in Syria, Jordan, Lebanon, Gaza, and the West Bank, with a prevalence of 12.1% for type 2 diabetes and 18.6% for hypertension among adults over 40 years old [10]. Treatment for these chronic diseases now consumes a substantial part of the healthcare system budgets of the United Nations (UN) agency responsible for support of this population [1].

Chronic diseases affecting long-term refugee settlements are commonly nutrition-related conditions such as diabetes and cardiovascular disease; in addition, >40% of refugees (and, in some “fields” [refugee settlement areas designated by the UN], >80%) receive food aid. This aid is primarily composed of grain, flour, and rice, under a traditional model that emphasizes maintenance of calorie intake rather than dietary diversity [11]. The reason for this composition of aid is 2-fold: first, because aid was traditionally designed for emergency situations to address acute under-nutrition needs and second, because of supply-side pressures to dump excess United States agricultural production on international markets through food aid [12]. Reducing chronic disease among refugees through modification of food aid packages for primary prevention has become a subject of intensive discussion and preliminary policy changes, particularly because healthcare systems are so under-resourced in the region that funding for secondary prevention is severely constrained [13,14]. At least three reforms have been proposed and piloted to different degrees in field-based trials: (i) partial replacement of traditional food parcel delivery (“in-kind” food aid) with electronic debit cards (“e-vouchers”) restricted to purchasing non-tobacco/alcohol foods [14–16], (ii) partial replacement of traditional food parcel delivery with unrestricted cash [14,17], and/or (iii) an alternative food parcel that replaces a portion of grain with fruits and vegetables (canned, dried, or—if locally available—fresh) [18–20].

How much reduction in chronic disease, if any, would be expected from the proposed food aid policy changes remains unclear because field-based trials or pilot demonstration projects cannot be sustained for sufficient periods to track long-term outcomes. Increased dietary diversity, food security, sense of agency, and local economic growth have been observed from field-based randomized trials and pilot demonstration projects of alternative food aid. Diversion towards non-food products, theft, corruption, or food price inflation have been minimal or undetected in these studies, even under the cash delivery policy [14–16,21]. Hence, agencies such as the World Food Program have begun to expand debit card or cash programs, despite opposition from the US government and associated private contractors invested in traditional food parcel delivery [22–24]. The World Food Program, World Health Organization, and others working with large refugee populations in the Middle East have called for simulation models to help estimate the longer-term chronic disease consequences of alternative food aid [14,20].

The principal aim of this study was to identify the potential changes in type 2 diabetes and cardiovascular disease among Palestinian refugees transitioning from traditional food aid to an alternative aid (debit card, cash, or alternative food parcel). Our primary a priori hypothesis was that transitioning from traditional food parcel delivery to a debit card for food would reduce the overall incidence and complications from type 2 diabetes and cardiovascular disease, given prior evidence that the debit card intervention was particularly effective at increasing measures of dietary diversity [15,16]. The secondary hypotheses were that the transition to debt card would be more effective than substitution to cash, but less effective than transition to an alternative food parcel.

## Methods

### Ethical approval

Approval for this study was obtained from the Institutional Review Board of Stanford University (eProtocol number IRB-39274).

Study design and reporting was based on the Modeling Good Research Practice Guidelines by the International Society for Pharmacoeconomics and Outcomes Research (ISPOR) [25]. S1 Text details the data underlying the results and provides the prospective analysis plan.

### Model overview

An individual-level microsimulation was created to estimate the impact of food aid delivery methods on food consumption, type 2 diabetes, and cardiovascular disease outcomes among the 20- to 79-year-old subpopulation receiving food aid (43%) among 5.3 million registered Palestinian refugees in Syria, Jordan, Lebanon, Gaza, and the West Bank. The simulation took into account the proportion who received food aid and the dietary patterns among them, including the portion of food acquired and consumed from other sources besides food aid. Individual people were simulated by sampling from the large datasets specified below to capture the correlations among demographic, food consumption, and cardiometabolic variables, including age, sex, nutrition profile, diabetes and cardiovascular disease biomarkers, chronic disease history, and medication use.

**Fig 1:**
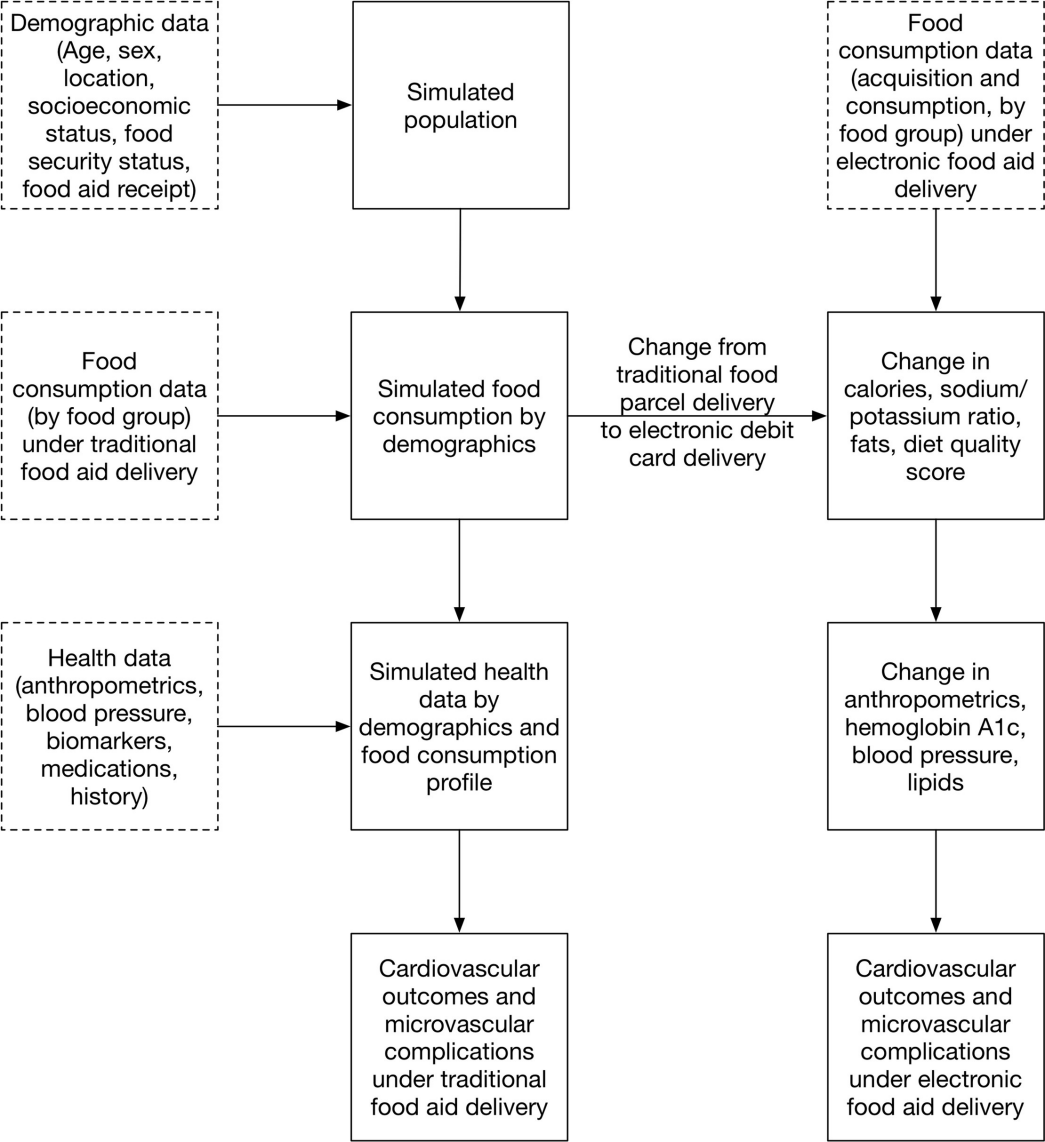
Model diagram. Data are in dashed squares and simulation steps in solid squares. See Tables 1 and 2 for data sources and parameter values.

The microsimulation (Fig 1) had the following 3 key components: (i) a demographic component specifying features of the refugee population as of 2017, including age, sex, and location; (ii) a food consumption component reflecting individual food consumption in major categories based on the person’s demographics, incorporating both aid-based and non–aid-based food, and conditional on whether the person received food parcel delivery or electronic debit card delivery of food aid; and (iii) a health component, reflecting both demographics and food consumption as well as detailing diabetes and cardiovascular disease history, biomarkers, medications, and the estimated probability of subsequent outcome events, listed below.

### Population data

Demographic data were obtained from the UN (2017) on 5,340,443 registered refugees in Syria, Jordan, Lebanon, Gaza, and the West Bank, including age, sex, and location (the 5 locations above; Table 1) [26].

**Table 1:** Demographic, food consumption, and health data from Palestinian refugees in the Middle East. Demographic data from the UN (*n* = 5,340,443; 2017), food consumption data from the World Food Program (*n* = 2,554 households; 2011–2017), and health data from UNRWA (*n* = 516,386; 2012–2015), specifically from the electronic health record that is believed to be comprehensive given universal refugee registration procedures that establish a population denominator for each estimate [15,16,26]. The data are from pilot studies conducted among the affected population in community-based settings to specifically address how much (or how little) responsiveness occurs in the context of food availability in refugee cities and towns.

Characteristic	Mean (IQR)
**Demographics**	
Registered refugees, no.	5,340,443
Proportion in each location, %	25.3% in Gaza, 15.1% in the West Bank, 10.2% in Syria, 8.7% in Lebanon, and 40.7% in Jordan
Age, years	32.5 (15.6–43.2)
Proportion female, %	60.1%
Proportion enrolled in food aid programs, %	43.0%
**Traditional food parcel delivery (“in-kind” food aid)**	
Total food consumption, kcal/person/day	2,296 (845–2,362)
Consumption of cereals, kcal/person/day	1,239 (147–2,331)
Consumption of tubers, pulses, legumes, and nuts, kcal/person/day	137 (7–266)
Consumption of fruits and vegetables, kcal/person/day	230 (9–452)
Consumption of animal products, kcal/person/day	343 (38–648)
Consumption of additional oils and fats, kcal/person/day	163 (95–230)
Consumption of sugars, kcal/person/day	184 (14–383)
**Electronic debit card food aid (“e-voucher”)**	
Total food consumption, kcal/person/day	2,441 (1,227–2,840)
Consumption of cereals, kcal/person/day	1,209 (1,125–1,292)
Consumption of tubers, pulses, legumes, and nuts, kcal/person/day	157 (15–300)
Consumption of fruits and vegetables, kcal/person/day	374 (32–715)
Consumption of animal products, kcal/person/day	423 (33–813)
Consumption of additional oils and fats, kcal/person/day	157 (127–188)
Consumption of sugars, kcal/person/day	178 (1–355)
**Cardiovascular biomarkers**	
BMI, kg/m^2^	27.1 (22.5–31.7) among men, 30.2 (25.4–35.2) among women
Systolic blood pressure, mmHg	131.7 (120.0–140.0) among men, 120.1 (110.0–130.0) among women
Diastolic blood pressure, mmHg	80.9 (74.0–88.0) among men, 74.7 (70.0–80.0) among women
Total cholesterol, mmol/L	187.0 (157.5–211.5) among men, 195.1 (165.0–218.0) among women
HDL cholesterol, mmol/L	38.6 (31.0–45.0) among men, 44.5 (36.0–50.0) among women
**Diagnostic history and treatment profile based on electronic health records**	
Proportion with type 2 diabetes mellitus, %	12.2%
Hemoglobin A1c among those with type 2 diabetes mellitus, %	8.2 (6.7–9.6)
Diabetes treatment, of those with diabetes, %	86.0%
Current tobacco smoking, %	25.1%
Serum creatinine, micromol/L	75.1 (67.3–83.0)
Urine microalbumin/creatinine ratio, mg/mmol	5.0 (3.7–6.3)
Blood pressure treatment, of those with hypertension, %	84.9%
Statin treatment, %	6.1%
Cardiovascular disease history, %	1.6%
Nephropathy prevalence among persons with diabetes, %	11.9%
Neuropathy prevalence among persons with diabetes, %	19.5%
Retinopathy prevalence among persons with diabetes, %	14.3%
All-cause mortality rate, per 1,000 person-years	3.0

**Abbreviations:** BMI, body mass index; HDL, high-density lipoprotein; IQR, interquartile range; kcal, kilocalories; UNRWA, United Nations Relief and Works Agency.

Food consumption data were obtained from the World Food Program (2011–2017) on registered refugees receiving food aid by the demographic variables listed above, categorized by quantity and caloric value of major food group procured and consumed per person per day, including aid and nonaid food (Table 1) [15,16]. Food procurement and consumption were separately tabulated for persons receiving traditional food parcel delivery of food aid (*n* = 1,507 households) and for persons obtaining electronic debit card delivery of food aid (*n* = 1,047 households), as detailed further below.

Health data were obtained from a random 10% sample of people in the electronic health record system of the UN Relief and Works Agency (UNRWA, the UN body responsible for care of registered refugees in Syria, Jordan, Lebanon, Gaza, and the West Bank, who are treated through 143 primary health facilities), stratified by age, sex, principal diagnosis, and location (2012–2015; *n* = 516,386; Table 1). The health data included the most recent value of age, sex, height, weight, systolic and diastolic blood pressure, hemoglobin A1c (percent), total and high-density lipoprotein (HDL) cholesterol (mmol/L), tobacco smoking status, serum creatinine, urine microalbumin-to-creatinine ratio, cardiovascular and metabolic medications (anticoagulants, antihypertensives, lipid therapies, and glycemic treatment), and diabetes and cardiovascular history (history of myocardial infarction or stroke).

### Outcome metrics

Outcome metrics included key outcomes considered strongly attributable to changes in nutrition per a recent systematic review and meta-analysis [27]: incidence per 1,000 person-years of hypertension (systolic blood pressure ≥130 mmHg or diastolic ≥80 mmHg or prescription of an antihypertensive medication), type 2 diabetes (hemoglobin A1c ≥6.5%, fasting plasma glucose ≥126 mg/dL, random plasma glucose ≥200 mg/dL, or prescription of a diabetes medication), atherosclerotic cardiovascular disease events (first nonfatal or fatal myocardial infarction or stroke), each of three microvascular complications (end-stage renal disease, diabetic neuropathy manifest as pressure sensation loss by 10 g monofilament test, and proliferative diabetic retinopathy leading to severe vision loss with Snellen test acuity <20/200), and all-cause mortality.

### Exposures

Two exposures were compared in the base case analysis: food parcel delivery of food aid and electronic debit card delivery of food aid. Food parcel delivery involved standardized monthly food parcels for each household that could be obtained by the designated recipient for each household at central locations in the community by participants. Each parcel contained wheat flour, white rice, sugar, vegetable oil, chickpeas, lentils, dried whole milk, and canned sardines. Electronic delivery involved $30 per household member per month in value (in 2017 US dollars; roughly the same value as the food parcel) added via automated bank transfer to an electronic debit card, which could be used with an individual PIN code at participating store locations in the community. Delivery was accompanied by a text message reminder to the assigned card bearer’s phone for each household. Debit cards were electronically restricted to permit food purchases, excluding chocolates, alcohol, cigarettes, soda, or non-food items [28]. Both exposures were directed to refugees with household income below the location-specific definitions of absolute (approximately $3.7/person/day) or abject poverty (approximately $1.5/person/day) by a means test, along with those enrolled in parallel aid programs or meeting other definitions of socioeconomic marginalization (e.g., elderly, widowed, and living alone) [29,30].

The change in food consumption attributable to a change from food parcel to electronic debit card delivery was estimated from a series of field-based studies by the World Food Program, comparing households after piloted roll-out of electronic debit card food aid to similar households remaining on food parcel delivery within each location (*n* = 2,554 households) [15,16]. Consumption in kilocalories (kcal) per person per day was obtained from quantitative household food frequency questionnaires among registered refugees, specifying daily per person consumption in each of 250 food categories (S1 Table). Major changes in consumption in each of the major Food and Agriculture Organization food groups (cereals; tubers, pulses, legumes, and nuts; fruits and vegetables; animal products; additional oils and fats (apart from animal products); and sugars [31]) are summarized in Table 1.

### Impact simulations

The estimated change in food consumption attributable to a change from food parcel to electronic debit card delivery was used to estimate changes in each outcome metric. Specifically, 4 intermediate variables were calculated to translate change in food consumption to change in each outcome metric: change in total calories, change in sodium and potassium intake, change in fatty acids (polyunsaturated, monounsaturated, and saturated fats), and change in overall dietary quality (Mediterranean Dietary Score [MDS] [32]). The MDS was chosen because it is predictive of incident type 2 diabetes independently from total calorie intake [33], and dietary interventions corresponding to MDS improvements have resulted in reduced type 2 diabetes incidence in prior trials among population in the Middle East [34,35]. Change in total calories was calculated by summing change in each food category from the field-based studies cited above. Change in sodium, potassium, and fatty acids were estimated based on the per-unit content estimates of each food from standardized international databases [36,37], with bootstrapping across the distribution of content variations within each food category to construct 95% confidence intervals (CIs) (S2 Table). Change in MDS was estimated by calculating the score from the food patterns reported in quantitative food frequency questionnaires (S1 Text).

Change in total calories was used to estimate changes in body mass index (BMI), using the National Institutes of Health/Hall model of body weight change, which conditions body weight change estimates on age, sex, height, and starting weight, based on results of randomized controlled trials (S1 Text) [38–40]. Changes in sodium and potassium were used to estimate changes in systolic blood pressure using regression models from a large, international prospective epidemiological study (S1 Text) [41]. Changes in fatty acids were used to estimate changes in total and HDL cholesterol using regression models from a meta-analysis of randomized controlled trials (S1 Text) [42]. Changes in BMI and MDS were used to estimate change in hemoglobin A1c based on regression models from meta-analyses of prospective studies and risk projections (S1 Text) [43,44].

The health implications of the change from food parcel to electronic debit card delivery of food aid were estimated among a simulated refugee population of 5.3 million people. To produce the simulated population, Monte Carlo sampling with copulas was used [45], which is a strategy to repeatedly sample with replacement from the distribution of each variable in the demographic and health datasets: baseline age, sex, height, weight, systolic and diastolic blood pressure, hemoglobin A1c (percent), total and HDL cholesterol (mmol/L), tobacco smoking status, serum creatinine, urine microalbumin-to-creatinine ratio, cardiovascular and metabolic medications, and diabetes and cardiovascular history as of 2015. When sampling, the approach of Monte Carlo sampling with copulas captures both the marginal distribution of each factor as well as the correlations among the factors (S1 Fig).

Following current modeling guidelines [46], the full life course of the population was simulated for any persons alive during a 10-year policy planning horizon (2016–2025). The outcome metrics among the population were simulated with traditional food aid delivery (baseline simulation), and then after transition to electronic debit card delivery of food aid (intervention simulation). In each simulation, UN birth rate estimates were used to simulate new births, the age- and sex-specific linear secular trend in each biomarker was used to simulated biomarker changes over time in each population, and both cardiovascular and all-cause mortality equations calibrated to the death statistics at each location were used to estimate deaths conditional on demographics and biomarkers [47]. Incidence of type 2 diabetes and hypertension were defined each year of the simulation based on blood pressure and hemoglobin A1c, respectively [48,49]. The incidence of cardiovascular disease events—first nonfatal or fatal myocardial infarction, first nonfatal or fatal stroke—was estimated from the Globorisk equations for each location, which used age, sex, smoking status, diabetes status, systolic blood pressure, and total cholesterol to estimate risk [50,51]. The incidence of each of three microvascular complications—end-stage renal disease, diabetic neuropathy, and diabetic retinopathy—and all-cause mortality were estimated by recalibrating the baseline hazard rate in the Risk Equations for Complications of Type 2 Diabetes (RECODe) to the observed rate of each complication from the most recent UNRWA health audits (Table 1) [52,53]. To account for uncertainty in the effect sizes of diet on health outcomes, we bootstrapped across the distribution of 95% CIs around each coefficient in the input equations to construct 95% CIs around each outcome metric. We note these estimation approaches attempt to focus on previously validated physiological models of causality from specific nutritional components to the outcomes of interest, at the risk of underestimating indirect correlational effects for which causal pathways are not yet clearly established.

As an alternative to the debit card program, we simulated 2 other potential interventions. First, we simulated transitioning from debit card delivery of food aid to transitioning to cash aid, which has been introduced at several sites. The change in food consumption attributable to a change from food parcel to cash aid was estimated by food frequency questionnaires in a field-based study (*n* = 1,041 households receiving a debit card compared to 1,507 receiving cash aid) and included analysis of diversion of cash for non-food uses [14].

Second, we simulated a proposed alternative food parcel that would reduce the amount of grain within the food parcel but increase the amount of fruits and vegetables (canned, dried, and—where possible—fresh) included by way of both regional and local sourcing [20,24]. We specifically performed a threshold analysis to estimate how much decrease in grain and increase in fruits and vegetables would be needed to reduce the rate of the chronic disease outcomes in our model without increasing the total aid budget, given the exchange rate in budgetary dollars of bundled grain costs versus bundled fruit and vegetable costs. The latter costs incorporated the additional procurement, labor, transport, and storage costs of increased fruit and vegetable inclusion; correction for seasonal price variations; and (for noncanned/nondried fresh fruits and vegetables) additional refrigerated transport and storage and increased delivery frequency to avoid spoilage (Table 1) [54,55]. We restricted the threshold analysis to ensure that the lower 95% CI bounds of total available food per person per day from the alternative food parcel would always remain above the World Health Organization/Food and Agricultural Organization goals to avoid food insecurity (>2,100 kcal/person/day, >10% from protein, >17% from fat [11]).

### Sensitivity analyses

#### Mis-reporting biases

In a sensitivity analysis, we re-estimated differential outcomes in all 3 scenarios (transition to debit card, cash delivery, or alternative food parcel) when sampling from the empirical distributions of observed bias in how much food frequency questionnaires may under- or overestimate true consumption (S3 Table). We adjusted consumption estimates from the food frequency questionnaires to correct for under- or overconsumption based on comparisons of food frequency questionnaire responses to 12-day weighted food records over a 12-month period, which estimated typically 47.6 kcal/person/day (SE: 0.8 kcal/person/day) underreporting of cereal consumption, 2.8 kcal/person/day (SE: 0.2) overreporting for tubers, 257.5 kcal/person/day (SE: 3.4) overreporting for fruits and vegetables, 2.1 kcal/person/day (SE: 1.1) underreporting for animal products, 20.4 kcal/person/day (SE: 0.1) underreporting for oils and fats, and 1.2 kcal/person/day (SE: 0.1) underreporting for sugars [56].

#### Compensatory behaviors

In a second sensitivity analysis, specific to the alternative food parcel simulation, we estimated the degree of compensatory increase in nonaid food consumption—particularly of refined grains, oils and fats, and confectionaries—that would be necessary from out-of-pocket food purchases to fully neutralize any benefits of the alternative food parcel.

#### Enhanced aid budget

In a third sensitivity analysis, also specific to the alternative food parcel simulation, we estimated the degree of increase in funding for the parcel that would be necessary to achieve a 1.0-point absolute increase in the MDS, which is the point increase associated with a clinically meaningful improvement in cardiovascular disease event incidence (myocardial infarction and stroke) [57].

### Cost-effectiveness analysis

Following recently updated cost-effectiveness guidelines [46], the incremental cost-effectiveness of transitioning away from traditional food parcel delivery of food aid to alternative strategies (debit card, cash, or alternative food parcel) was calculated from both a healthcare system and a societal perspective (S4 Table). Costs from each of the healthcare and societal perspectives are itemized in Table 2 and were computed from the same life-course perspective over a 10-year policy planning horizon, as with the impact simulation above. Costs for both delivery mechanisms included formal and informal healthcare sector expenditures (reflecting observed trends in service provision, pharmaceutical dispensation, and utilization at UNRWA facilities), and nonhealthcare expenditures (including overhead expenditure and costs of in-kind food or debit card expenditures). Disability-adjusted life-years (DALYs; the sum of years of life lost and utility-weighted years of life lived with disability) were calculated based on utility weights for each outcome metric from a prior international survey (Table 2) [58]. Both costs and DALYs were discounted at a 3% annual rate, and 2 incremental cost-effectiveness ratios (one for the healthcare sector perspective, the other for the societal perspective) were expressed in 2017 US dollars per DALY.

**Table 2:** Input parameters for estimating the cost-effectiveness of shifting from traditional food parcel delivery (“in-kind” food aid) to electronic debit card delivery of food aid (“e-vouchers”). Costs are in 2017 US dollars and reflect negotiated prices for UN Relief and Work Agency facilities, including out-of-pocket costs to patients and costs absorbed by UN facilities. See S2 Table for impact inventory. Health utilities refer to the degree of disability on a scale from 0 to 1 conferred by a given disease (with larger disutilities indicating more disabling disease, and 1 indicating death).

Parameter	Mean (95% CI)
**Health state utilities**	
Cardiovascular disease events (myocardial infarction or stroke)	0.28 (0.02–0.58)
End-stage renal disease	0.57 (0.40–0.75)
Diabetic neuropathy	0.10 (0.07–0.13)
Diabetic retinopathy	0.19 (0.13–0.25)
**Healthcare costs**	
Blood pressure treatment, per patient per year	$6.42 ($5.69–$7.15)
Diabetes treatment, per patient per year	$14.14 ($7.72–$35.55)
Lipid treatment, per patient per year	$5.96 ($4.72–$7.20)
Management of atherosclerotic cardiovascular disease events, per patient per event	$11,663.46 ($8,170.22–$15,143.86)
Management of end-stage renal disease, per patient per year	$14,128.68 ($12,896.10–$15,365.10)
Management of diabetic neuropathy, per patient per year	$995.26 (430.67–2,421.47)
Management of diabetic retinopathy, per patient per year	$3,519.97 ($665.95–$6,364.90)
**Food aid costs**	
Food parcel food material costs, per recipient per month	$37.00 ($31.00–$43.00)
Food parcel overhead and infrastructure costs, per recipient per month	$11.50 ($9.64–$13.36)
Electronic debit card food costs, per recipient per month	$30.00 ($13.50–$47.00)
Electronic debit card overhead and infrastructure costs, per recipient per month	$3.30 ($2.77–$3.83)
Relative costs of fruits and vegetables per pound versus grain per pound for procurement (ratio) (per kilogram)	$0.58 ($0.30–$1.20) ($1.29 [$0.67–$2.67])
Additional food parcel overhead and infrastructure costs under alternative food parcel with more fruits and vegetables, per recipient per month	$1.16 ($0.90–$1.80)

**Abbreviations:** CI, confidence interval; UN, United Nations.

### Uncertainty analysis

Uncertainty analysis was performed by Monte Carlo sampling with replacement 10,000 times from the probability distribution of all input parameters listed in the Tables, from which 95% CIs were calculated [59]. Analyses were weighted by frequency weights estimated to convert the healthcare input data to a representative Palestinian population sample (S5 Table). Simulations were performed in R (version 3.3.3; The R Foundation for Statistical Computing, Vienna, Austria) with the MASS [60] and tidyverse [61] packages, using the code uploaded at https://sdr.stanford.edu concurrent with publication.

## Results

### Population data

Demographic data from the UN indicated that, among the 5,340,443 registered refugees in Gaza, West Bank, Syria, Lebanon, and Jordan, the mean age was 32.5 years old (interquartile range [IQR] 15.6–43.2 years), with 60.1% female and 25.3% located in Gaza, 15.1% in the West Bank, 10.2% in Syria, 8.7% in Lebanon, and 40.7% in Jordan; 43.0% were enrolled in food aid programs (Table 1).

Food consumption data from the World Food Program among the population of refugees receiving traditional food parcel delivery of food aid averaged 2,296 kcal/person/day (IQR 845–2,362), including 1,239 kcal/person/day from cereals; 137 kcal/person/day from tubers, pulses, legumes, and nuts; 230 kcal/person/day from fruits and vegetables; 343 kcal/person/day from animal products; 163 kcal/person/day from additional/added oils and fats; and 184 kcal/person/day from sugars. Food consumption among the population of refugees receiving electronic debit card delivery of food aid averaged 2,441 kcal/person/day (IQR 1,258–3,623), including 1,209 kcal/person/day from cereals; 100 kcal/person/day from tubers, pulses, legumes, and nuts; 271 kcal/person/day from fruits and vegetables; 526 kcal/person/day from animal products; 157 kcal/person/day from additional oils and fats; and 178 kcal/person/day from sugars (Table 1).

Health data from UNRWA indicated a mean BMI of 29.2 kg/m^2^ (IQR 23.5–34.1), a mean systolic blood pressure of 124.6 mmHg (IQR 111.7–136.5), a mean diastolic blood pressure of 76.5 mmHg (IQR 66.7–85.3), a mean total cholesterol of 4.9 mmol/L (IQR 4.6–5.2), a mean HDL cholesterol of 1.1 mg/dL (IQR 0.8–1.4), and a current prevalence of 12.2% for type 2 diabetes mellitus, with people with type 2 diabetes mellitus averaging a hemoglobin A1c of 8.2% (IQR 6.7–9.6) (Table 1). We note that the UNRWA data cover all locations with Palestinian refugees (not only the West Bank and Gaza but also the fields in Syria, Jordan, and Lebanon).

### Impact simulations

We did not find that the change from food parcel to electronic debit card delivery of food aid led to a meaningful change in consumption, biomarkers, or disease outcomes in our simulation model (Table 3). Among those receiving food aid in our model, the change from food parcel to electronic debit card delivery of food aid was estimated to produce an overall 145 kcal/person/day increase in calorie consumption (95% CI 647 decrease to 929 increase, from the baseline of 2,296), 641 mg/person/day increase in sodium consumption (95% CI 2,321 decrease to 2,793 increase, from a baseline of 4,288), 258 mg/person/day increase in potassium consumption (95% CI 2,277 decrease to 3,102 increase, from a baseline of 3,834), 2,014 mg/person/day increase in saturated fatty acid consumption (95% CI 7,507 decrease to 11,429 increase, from a baseline of 26,124), 2,689 mg/person/day increase in monounsaturated fatty acid consumption (95% CI 10,039 decrease to 15,293 increase, from a baseline of 28,567), 938 mg/person/day increase in polyunsaturated fatty acid consumption (95% CI 5,860 decrease to 7,657 increase, from a baseline of 26,871), and a 0.1 point increase in the MDS on a scale from 0 to 14, such that higher values indicate better adherence to the Mediterranean diet (95% CI −1.0 to 1.0, from a baseline of 7.9).

**Table 3:** Estimated changes in nutrition from a change in food aid. Model-derived estimates of changes in dietary intake measures attributable to a change from food parcel to electronic debit card delivery of food aid; a change from food parcel to cash aid; or a change from food parcel to alternative food parcel with less grain and increased fruit and vegetable content.

Change in consumption, per person per day	Mean change (95% CI) attributable to change in aid		
Change from traditional (in-kind) food aid to:	Debit card	Cash	Alternative parcel
Calories (kcal)	+145 (−647 to +929)	+238 (−603 to +1,077)	−33 (−27 to +38)
Sodium (mg)	+641 (−2,321 to +2,793)	+865 (−2,236 to +3,861)	−64 (−68 to −60)
Potassium (mg)	+258 (−2,277 to +3,102)	+517 (−2,194 to +3,179)	−1 (−18 to +15)
Saturated fatty acid (mg)	+2,014 (−7,507 to +11,429)	+2,773 (−7,407 to +12,840)	+345 (+340 to +350)
Monounsaturated fatty acid (mg)	+2,689 (−10,039 to +15,293)	+3,779 (−9,837 to +17,268)	+358 (+349 to +367)
Polyunsaturated fatty acid (mg)	−938 (−5,860 to +7,657)	+1,588 (−5,623 to +8,728)	+342 (+336 to +347)
MDS (absolute scale)	+0.1 (−1.0 to +1.0)	+0.1 (−1.0 to +1.0)	+0.2 (0 to +2.0)

**Abbreviations:** CI, confidence interval; MDS, Mediterranean Dietary Score.

The changes in food consumption among those receiving food aid, attributable to the change from food parcel to electronic debit card delivery of food aid, were estimated by our model to produce a 2.5 kg/m^2^ increase in BMI (95% CI 11.4 decrease to 17.0 increase, from a baseline of 29.2), 0.58 mmHg increase in systolic blood pressure (95% CI 5.61 decrease to 6.81 increase, from a baseline of 124.6), 0.29 mmHg increase in diastolic blood pressure (95% CI 2.14 decrease to 2.71 increase, from a baseline of 76.5), 0.06 mmol/L increase in total cholesterol (95% CI 0.17 decrease to 0.29 increase, from a baseline of 4.9), and a 0.11 mmol/L increase in HDL cholesterol (95% CI 0.55 decrease to 0.75 increase, from a baseline of 1.1; Table 4).

**Table 4:** Estimated changes in biomarkers from a change in food aid. Model-derived estimates of changes in dietary intake measures attributable to a change from food parcel to electronic debit card delivery of food aid; a change from food parcel to cash aid; or a change from food parcel to alternative food parcel with less grain and increased fruit and vegetable content.

Change in biomarkers	Mean change (95% CI) attributable to change in aid		
Change from traditional (in-kind) food aid to:	Debit card	Cash	Alternative parcel
BMI (kg/m^2^)	+2.5 (−11.4 to +17.0)	+4.2 (−10.6 to +19.8)	−0.6 (−0.8 to −0.4)
Systolic blood pressure (mmHg)	+0.58 (−5.61 to +6.81)	+0.35 (−6.15 to +6.89)	−0.12 (−0.16 to −0.08)
Diastolic blood pressure (mmHg)	+0.29 (−2.14 to +2.71)	+0.23 (−2.33 to +2.77)	−0.05 (−0.07 to −0.03)
Total cholesterol (mmol/L)	+0.06 (−0.17 to +0.29)	+0.07 (−0.17 to +0.32)	−0.004 (−0.014 to +0.006)
HDL cholesterol (mmol/L)	+0.11 (−0.55 to +0.75)	+0.17 (−0.53 to +0.85)	−0.03 (−0.04 to −0.02)

**Abbreviations:** BMI, body mass index; CI, confidence interval; HDL, high-density lipoprotein.

The changes in biomarkers, attributable to the change from food parcel to electronic debit card delivery of food aid, were estimated by our model to produce a 0.14 per 1,000 person-years increase in the incidence of hypertension (95% CI 3.03 decrease to 2.61 increase, from a baseline of 35.06), 0.77 per 1,000 person-years increase in the incidence of type 2 diabetes (95% CI 3.14 decrease to 6.98 increase, from a baseline of 12.24), 0.40 per 1,000 person-years increase in the incidence of atherosclerotic cardiovascular disease events (95% CI 1.03 decrease to 1.14 increase, from a baseline of 6.58), 0.11 per 1,000 person-years incidence in the incidence of end-stage renal disease (95% CI 0.34 decrease to 0.72 increase, from a baseline of 1.56), 0.24 per 1,000 person-years incidence in the incidence of diabetic neuropathy (95% CI 1.17 decrease to 2.10 increase, from a baseline of 4.41), 0.36 per 1,000 person-years incidence in the incidence of proliferative diabetic retinopathy (95% CI 1.52 decrease to 2.66 increase, from a baseline of 4.53), and 0.16 increase per 1,000 person-years all-cause mortality (95% CI 0.51 decrease to 1.11 increase, from a baseline of 3.50) among those receiving food aid (Table 5).

**Table 5:** Primary outcome estimates. Model-derived estimates of changes in chronic disease outcome measures attributable to a change from food parcel to electronic debit card delivery of food aid; a change from food parcel to cash aid; or a change from food parcel to alternative food parcel with less grain and increased fruit and vegetable content.

Outcome, per 1,000 person-years	Mean change (95% CI) attributable to change in aid		
Change from traditional (in-kind) food aid to:	Debit card	Cash	Alternative parcel
Hypertension incidence	+0.14 (−3.03 to +2.61)	+0.04 (−3.33 to +2.60)	−0.08 (−0.11 to −0.05)
Type 2 diabetes incidence	+0.77 (−3.14 to +6.98)	+1.28 (−2.96 to +8.05)	−0.18 (−0.22 to −0.14)
Cardiovascular disease events (myocardial infarction or stroke)	+0.40 (−1.03 to +1.14)	+0.52 (−0.94 to +1.31)	−0.18 (−0.19 to −0.17)
End-stage renal disease	+0.11 (−0.34 to +0.72)	+0.22 (−0.33 to +0.84)	−0.14 (−0.24 to −0.04)
Diabetic neuropathy	+0.24 (−1.17 to +2.10)	+0.43 (−1.09 to +2.42)	−0.05 (−0.08 to −0.03)
Diabetic retinopathy	+0.36 (−1.52 to +2.66)	+0.60 (−1.45 to +3.05)	−0.08 (−0.11 to −0.05)
All-cause mortality	+0.16 (−0.51 to +1.11)	+0.26 (−0.45 to +1.31)	−0.02 (−0.04 to −0.01)

**Abbreviation:** CI, confidence interval.

### Cash aid

Among those receiving food aid in our model, a change from food parcel to cash aid was estimated to produce an overall 238 kcal/person/day increase in calorie consumption (95% CI 603 decrease to 1,077 increase, from the baseline of 2,296), 865 mg/person/day increase in sodium consumption (95% CI 2,236 decrease to 3,861 increase, from a baseline of 4,288), 517 mg/person/day increase in potassium consumption (95% CI 2,194 decrease to 3,179 increase, from a baseline of 3,834), 2,773 mg/person/day increase in saturated fatty acid consumption (95% CI 7,407 decrease to 12,840 increase, from a baseline of 26,124), 3,779 mg/person/day increase in monounsaturated fatty acid consumption (95% CI 9,837 decrease to 17,268 increase, from a baseline of 28,567), 1,588 mg/person/day increase in polyunsaturated fatty acid consumption (95% CI 5,623 decrease to 8,728 increase, from a baseline of 26,871), and a 0.1 point increase in the MDS on a scale from 0 to 14 such that higher values indicate better adherence to the Mediterranean diet (95% CI −1.0 to 1.0, from a baseline of 7.9).

The changes in food consumption among those receiving food aid, attributable to the change from food parcel to cash aid, were estimated by our model to produce a 4.2 kg/m^2^ increase in BMI (95% CI 10.6 decrease to 19.8 increase, from a baseline of 29.2), 0.35 mmHg increase in systolic blood pressure (95% CI 6.15 decrease to 6.89 increase, from a baseline of 124.6), 0.23 mmHg increase in diastolic blood pressure (95% CI 2.33 decrease to 2.77 increase, from a baseline of 76.5), 0.07 mmol/L increase in total cholesterol (95% CI 0.17 decrease to 0.32 increase, from a baseline of 4.9), and a 0.17 mmol/L increase in HDL protein cholesterol (95% CI 0.53 decrease to 0.85 increase, from a baseline of 1.1).

The changes in biomarkers, attributable to the change from food parcel to cash aid, were estimated by our model to produce a 0.04 per 1,000 person-years increase in the incidence of hypertension (95% CI 3.33 decrease to 2.60 increase, from a baseline of 35.06), 1.28 per 1,000 person-years increase in the incidence of type 2 diabetes (95% CI 2.95 decrease to 8.05 increase, from a baseline of 12.24), 0.52 per 1,000 person-years increase in the incidence of atherosclerotic cardiovascular disease events (95% CI 0.94 decrease to 1.31 increase, from a baseline of 6.58), 0.22 per 1,000 person-years increase in the incidence of end-stage renal disease (95% CI 0.33 decrease to 0.84 increase, from a baseline of 1.56), 0.43 per 1,000 person-years increase in the incidence of diabetic neuropathy (95% CI 1.09 decrease to 2.42 increase, from a baseline of 4.41), 0.60 per 1,000 person-years increase in the incidence of proliferative diabetic retinopathy (95% CI 1.45 decrease to 3.05 increase, from a baseline of 4.53), and 0.26 increase per 1,000 person-years all-cause mortality (95% CI 0.45 decrease to 1.31 increase, from a baseline of 3.50) among those receiving aid (Table 5).

### Alternative food parcel

We observed that a food aid parcel would need to divert 8.5% of the current grain parcel to fruits and vegetables to reduce all chronic disease outcome measures while maintaining the same total aid budget, after accounting for uncertainty in fruit and vegetable procurement and delivery costs. In particular, such a shift would be expected to reduce supply of grain in food aid parcels by 40 kcal/person/day and increase fruit and vegetable supply by 7 kcal/person/day in food aid parcels. If the supply were fully consumed and did not result in any shift in non–aid-based food consumption, the change from the traditional to alternative food parcel would be expected to produce an overall 33 kcal/person/day decrease in calorie consumption (95% CI 27–38, from the baseline of 2,296), 64 mg/person/day decrease in sodium consumption (95% CI 60–68, from a baseline of 4,288), 1.3 mg/person/day decrease in potassium consumption (95% CI 18 decrease to 15 increase, from a baseline of 3,834), 345 mg/person/day increase in saturated fatty acid consumption (95% CI 340–350, from a baseline of 26,124), 358 mg/person/day increase in monounsaturated fatty acid consumption (95% CI 349–367, from a baseline of 28,567), 342 mg/person/day increase in polyunsaturated fatty acid consumption (95% CI 336–347, from a baseline of 26,871), and a 0.2 point increase in the MDS on a scale from 0 to 14 such that higher values indicate better adherence to the Mediterranean diet (95% CI 0.0–2.0, from a baseline of 7.9).

The changes in food consumption among those receiving food aid, attributable to the change from traditional food parcel to alternative parcel, were estimated by our model to produce a 0.57 kg/m2 decrease in BMI (95% CI 0.37–0.84, from a baseline of 29.2), 0.12 mmHg decrease in systolic blood pressure (95% CI 0.08–0.16, from a baseline of 124.6), 0.05 mmHg decrease in diastolic blood pressure (95% CI 0.03–0.07 from a baseline of 76.5), 0.004 mmol/L decrease in total cholesterol (95% CI 0.014 decrease to 0.006 increase, from a baseline of 4.9), and a 0.03 mmol/L decrease in HDL cholesterol (95% CI 0.02–0.04, from a baseline of 1.1).

The changes in biomarkers, attributable to the change from traditional food parcel to alternative parcel, were estimated by our model to produce a 0.08 per 1,000 person-years decrease in the incidence of hypertension (95% CI 0.05–0.11, from a baseline of 35.06), 0.18 per 1,000 person-years decrease in the incidence of type 2 diabetes (95% CI 0.14–0.22 from a baseline of 12.24), 0.18 per 1,000 person-years decrease in the incidence of atherosclerotic cardiovascular disease events (95% CI 0.17–0.19, from a baseline of 6.58), 0.14 per 1,000 person-years decrease in the incidence of end-stage renal disease (95% CI 0.04–0.24, from a baseline of 1.56), 0.05 per 1,000 person-years decrease in the incidence of diabetic neuropathy (95% CI 0.03–0.08, from a baseline of 4.41), 0.08 per 1,000 person-years decrease in the incidence of proliferative diabetic retinopathy (95% CI 0.05–0.11, from a baseline of 4.53), and 0.02 decrease per 1,000 person-years all-cause mortality (95% CI 0.01 decrease to 0.04 increase, from a baseline of 3.50) among those receiving aid (Table 5).

### Sensitivity analyses

#### Mis-reporting biases

When we adjusted for potential mis-reporting biases in food frequency questionnaires, the results of our comparative analyses were not qualitatively different from those of the primary analysis (S3 Table).

#### Compensatory behavior

In the alternative food parcel simulation, we estimated that only a small degree of compensatory increase in nonaid food consumption—particularly of refined grains, oils and fats, and confectionaries—would be necessary from out-of-pocket food purchases to fully neutralize any benefits of the alternative food parcel. If refugees receiving the alternative food parcel equally increased their consumption of all 3 categories of refined grains, oils and fats, and confectionaries by only 2.1%, the cardiometabolic benefits of the alternative food parcel shown in Table 5 would be fully neutralized (95% CIs crossing 0).

#### Enhanced aid budget

By contrast, we estimated that an increase in the alternative food parcel budget total aid budget (including procurement and overhead costs) by 27% (an additional $10.40 per person per month) would be necessary to meet the goal of an average 1.0-point absolute increase in the MDS. This increase would be accompanied by a 1.73 per 1,000 person-years decrease in the incidence of hypertension (95% CI 0.87–2.59, from a baseline of 35.06), 4.49 per 1,000 person-years decrease in the incidence of type 2 diabetes (95% CI 1.34–5.83 from a baseline of 12.24), 1.25 per 1,000 person-years decrease in the incidence of atherosclerotic cardiovascular disease events (95% CI 0.61–1.89, from a baseline of 6.58), 0.62 per 1,000 person-years decrease in the incidence of end-stage renal disease (95% CI 0.30–0.94, from a baseline of 1.56), 0.55 per 1,000 person-years decrease in the incidence of diabetic neuropathy (95% CI 0.26–0.81, from a baseline of 4.41), 1.74 per 1,000 person-years decrease in the incidence of proliferative diabetic retinopathy (95% CI 1.23–2.25, from a baseline of 4.53), and 0.55 decrease per 1,000 person-years all-cause mortality (95% CI 0.16–1.02, from a baseline of 3.50) among those receiving aid.

### Cost-effectiveness analysis

Because transitioning from food parcel to either electronic debit card delivery or cash aid delivery of food aid was not effective at improving the chronic disease outcomes, we restricted the cost-effectiveness analysis to examining the transition from food parcels to alternative food parcels. The changes in health outcomes—attributable to a 10-year policy change from food parcels to alternative food parcels—were estimated by our model to avert 3,034 discounted DALYs per 100,000 population, over the life courses of the simulated individuals both receiving and not receiving food aid (95% CI 2,532–3,617, from a baseline of 1,194,868 DALYs accumulated in the overall population; Table 6). From a healthcare sector perspective that included the 10-year costs of disease management but not the costs of the parcel food aid or debit card expenditures and infrastructure, the change from traditional to alternative food parcels was estimated to save $1,255,370 in discounted healthcare expenditures per 100,000 population (95% CI $1,047,389–$1,496,447, from a baseline of $150,631,285 in expenditures for the overall population; Table 6), including both out-of-pocket and healthcare delivery system costs. From a societal perspective that included the 10-year cost savings of traditional to alternative food parcel procurement and delivery, the cost-effectiveness of alternative food parcel was estimated to save the same amount, $1,255,370, because (by design) the alternative food parcel was designed to have the same overall food aid budget in discounted expenditures per 100,000 population (including overhead), from a baseline of $336,848,228 when including food aid and associated overhead costs (Table 6). The change from traditional to alternative food parcels was estimated to have an incremental cost-effectiveness ratio of $414 saved per DALY averted from either a healthcare sector or a societal perspective (95% CI $290–$591). If the alternative food parcel was increased in funding level by 27% (as in the “enhanced aid budget” scenario described above), the incremental cost-effectiveness ratio increased to $70,223 spent per DALY averted (95% CI $58,223–$121,746).

**Table 6:** Cost-effectiveness analysis. The table shows DALYs and costs (in 2017 US dollars) averted by shifting from traditional food parcel delivery to a food parcel with less grain and increased fruits and vegetables. The parcel has the same total food aid costs, including food material and overhead costs for procurement and delivery. DALYs and costs are discounted at a 3% annual rate and reflect the life-course DALYs averted per 100,000 population from a 10-year policy change.

Outcome estimate	Mean (95% CI), per 100,000 population
**DALYs averted from…**	
Cardiovascular disease events	2,809 (2,344–3,349)
End-stage renal disease	204 (171–243)
Diabetic neuropathy	3 (2–4)
Diabetic retinopathy	18 (15–21)
*Total DALYs averted*	3,034 (2,532–3,617)
**Healthcare costs averted from…**	
Blood pressure treatment	$493 ($411–$588)
Diabetes treatment	$64,625 ($53,918–$77,035)
Lipid treatment	$519 ($433–$618)
Management of atherosclerotic cardiovascular disease events	$653,103 ($544,902–$778,523)
Management of end-stage renal disease	$495,007 ($412,999–$590,067)
Management of diabetic neuropathy	$3,582 ($2,989–$4,270)
Management of diabetic retinopathy	$38,041 ($31,739–$45,346)
**Food aid costs averted from…**	
Food material, overhead and infrastructure costs	$0 ($0–$0) (by design)
*Total societal (healthcare + food aid) costs averted*	$1,255,370 ($1,047,389–$1,496,447)

*Negative numbers indicate increased costs in the new alternative food parcel scenario compared with the traditional food parcel aid scenario.

**Abbreviations:** CI, confidence interval; DALY, disability-adjusted life-year.

## Discussion

Contrary to the supposition in the literature, our findings do not robustly support the theory that transitioning from traditional food aid to either debit card or cash delivery alone would necessarily reduce chronic disease outcomes. Rather, an alternative food parcel would be more effective, even after matching current budget ceilings. But compensatory increases in consumption of less healthy foods may neutralize the improvements from an alternative food parcel unless total aid funding were increased substantially.

In this study, we created a simulation model for forecasting changes in type 2 diabetes and cardiovascular disease incidence and complications from a transition from traditional food aid to an alternative aid format (debit card, cash, or alternative food parcel) among Palestinian refugees. One contribution of simulation models is to quantify the degree of certainty or uncertainty in making a policy decision based on available evidence. We incorporated the most comprehensive data available from the comprehensive healthcare provider for Palestinian refugees and from the main food aid providers among Palestine refugees in the Middle East; by repeatedly sampling from these data sources, we rejected our primary a priori hypothesis that transitioning from traditional food parcel delivery to a debit card for food would reduce the overall incidence and complications from type 2 diabetes and cardiovascular disease. Contrary to our hypothesis and to the supposition in the literature [22–24], we observed large variation in the possible trajectories of chronic disease from the changes in diet observed among refugees receiving alternative food aid delivery strategies, spanning the spectrum from both increased to decreased chronic disease morbidity and mortality. This addresses the request from policymakers to understand the degree to which proxy outcomes observed from short-term field-based trials may translate into long-term chronic disease outcomes and costs, particularly accounting for uncertainties around the estimated results [14,20]. Our findings suggest that the dietary measures and observations in extant studies do not robustly support the idea that chronic disease incidence would necessarily be reduced by a transition from traditional food aid to either debit card or cash delivery alone.

We did find, however, that transitioning to an alternative food parcel could reduce chronic disease morbidity and mortality with less uncertainty in the projected outcomes if the alternative food parcel transitioned to slightly less grain and more fruits and vegetables. We found, in particular, that small changes at the individual level in cardiometabolic risk factors would be expected to translate into larger population-level health gains and cost savings given the high prevalence of these diseases. The magnitude of the gains are somewhat higher than that anticipated, for example, from general sodium reduction in the food supply [62]. The alternative food parcel would be expected to be both effective and cost-effective even if the total aid budget—including additional infrastructure costs for fruit and vegetable procurement and delivery—were unchanged. Uncertainty was lower in this simulation due in part to the narrowed set of food content available for the food aid parcel, as opposed to the large range of content available in local stores. Our analyses importantly accounted for the proportion of the overall refugee population who receive food aid and the dietary patterns among them, including the portion of food acquired and consumed from other sources besides food aid. The microsimulation was also strengthened by using individual participant data from large datasets capturing the correlations among demographic, food consumption, and cardiometabolic variables, including age, sex, nutrition profile, diabetes and cardiovascular disease biomarkers, chronic disease history, and medication use.

Our analysis has important caveats and limitations. There is much uncertainty around our estimates due to the task of modeling long-term outcomes from shorter-term trials while accounting for the wide variation in nutrient profiles of available foods and their cardiometabolic consequences. While we focused on the chronic disease implications of transitions in food aid, there are other important reasons why food aid transitions might be important—including improved agency and choice among refugees, stimulus to local economies in semipermanent or permanent settlements, and avoidance of international dumping of undesired or extra crops under the guise of food aid [12]. Importantly, the primary analysis assumed that the nonaid portion of food consumption would not change with the change in aid, such that people would not compensate for the change by buying more grain and less fruits or vegetables with their own limited income; this assumption must be tested in field studies. An additional limitation is that we focused on a narrow subset of nutritional outcomes—type 2 diabetes and cardiovascular disease risk factors and outcomes—for which validated equations are available to translate food intake estimates into outcomes [50, 52–53]. Nutrition plays an important role in additionally reducing cancers and other diseases and improving infant health, for which equations are not yet well established [62,63]. Furthermore, our results are based on food frequency questionnaires, which are known to be less reliable and subject to underreporting compared to 24-hour dietary recalls. Finally, it is important to note that interventions that are effective in providing nutritional support in many locales may not be effective in the Gaza Strip, where blockades, conflict, and funding crises can limit quantities of goods distributed through the UN and humanitarian organizations. Additionally, prior detailed qualitative studies suggest that the supply chains for locally produced food versus internationally supplied food must undergo substantial review and modification to achieve the aim of an alternative food aid intervention [64–67].

The next logical step following this analysis is to perform field-based randomized studies of the alternative food parcel to identify unanticipated barriers to achievement, the role of seasonality and spoilage, and the degree to which refugees do or do not alter their diets in the context of an alternative food parcel. Prior to such randomized studies, our findings do not robustly support the theory that transitioning from traditional food aid to either debit card or cash delivery alone would necessarily reduce chronic disease outcomes. We did find, however, that transitioning to an alternative food parcel could be effective and should be explored further.

## Supporting information

S1 Text Prospective analysis plan and model equations. (PDF)

S1 Table Estimated distribution of kcal consumed per person per day by food type, under traditional food parcel delivery versus electronic food aid delivery. (DOCX)

S2 Table Distribution of content variations by food category. (DOCX)

S3 Table Alternative results when sampling from the distribution of potential under- and overreporting by food class. (DOCX)

S4 Table Impact inventory for elements included and not included in the cost-effectiveness analysis of electronic debit card delivery of food aid (“e-vouchers”) versus traditional food parcel delivery (“in-kind” food aid). (DOCX)

S5 Table Demographics of the healthcare input data versus the Palestinian refugee population. (DOCX)

S6 Table Energy metabolism parameter values used in the model for body weight change from a change in caloric intake. (DOCX)

S7 Table Globorisk equation coefficients. (DOCX)

S8 Table RECODe equation coefficients. (DOCX)

S9 Table CHEERS checklist for the cost-effectiveness analysis. (DOCX)

S1 Fig Covariance between key demographic, dietary, and health features. (DOCX)

## Abbreviations

BMI: body mass index
CI: confidence interval
DALY: disability-adjusted life-year
HDL: high-density lipoprotein
IQR: interquartile range
ISPOR: International Society for Pharmacoeconomics and Outcomes Research
kcal: kilocalories
MDS: Mediterranean Dietary Score
RECODe: Risk Equations for Complications of Type 2 Diabetes
UN: United Nations
UNRWA: UN Relief and Works Agency

## References

[1] Shahin Y, Kapur A, Seita A. Diabetes care in refugee camps: the experience of UNRWA. Diabetes Res Clin Pract. 2015;108: 1–6. 10.1016/j.diabres.2015.01.035 25680680 10.1016/j.diabres.2015.01.035

[2] Hamdan M, Defever M. A ‘transitional’ context for health policy development: the Palestinian case. Health Policy. 2002;59: 193–207. 11823024 10.1016/s0168-8510(01)00174-9

[3] Gersh BJ, Sliwa K, Mayosi BM, Yusuf S. The epidemic of cardiovascular disease in the developing world: global implications. Eur Heart J. 2010;31: 642–648. 10.1093/eurheartj/ehq030 20176800 10.1093/eurheartj/ehq030

[4] Husseini A, Abu-Rmeileh NM, Mikki N, Ramahi TM, Ghosh HA, Barghuthi N, et al Cardiovascular diseases, diabetes mellitus, and cancer in the occupied Palestinian territory. Lancet. 2009;373: 1041–1049. 10.1016/S0140-6736(09)60109-4 19268350 10.1016/S0140-6736(09)60109-4

[5] Amara AH, Aljunid SM. Noncommunicable diseases among urban refugees and asylum-seekers in developing countries: a neglected health care need. Global Health. 2014;10: 24 10.1186/1744-8603-10-24 24708876 10.1186/1744-8603-10-24 PMC3978000

[6] Yanni EA, Naoum M, Odeh N, Han P, Coleman M, Burke H. The Health Profile and Chronic Diseases Comorbidities of US-Bound Iraqi Refugees Screened by the International Organization for Migration in Jordan: 2007–2009. J Immigr Minor Heal. 2013;15: 1–9. 10.1007/s10903-012-9578-6 22307545 10.1007/s10903-012-9578-6

[7] Mokdad AH. Burden of cardiovascular diseases in the Eastern Mediterranean Region, 1990–2015: findings from the Global Burden of Disease 2015 study. Int J Public Health. 2017; 10.1007/s00038-017-1012-3 28776245 10.1007/s00038-017-1012-3 PMC5973984

[8] Mokdad AH. Burden of obesity in the Eastern Mediterranean Region: findings from the Global Burden of Disease 2015 study. Int J Public Health. 2017; 10.1007/s00038-017-1002-5 28776243 10.1007/s00038-017-1002-5 PMC5973977

[9] Mokdad AH. Diabetes mellitus and chronic kidney disease in the Eastern Mediterranean Region: findings from the Global Burden of Disease 2015 study. Int J Public Health. 2017; 10.1007/s00038-017-1014-1 28776240 10.1007/s00038-017-1014-1 PMC5973961

[10] United Nations Relief and Works Agency. Health Department Annual Report. Geneva: United Nations Relief and World Agency; 2018. Available from: https://www.unrwa.org/sites/default/files/content/resources/health_programme_annual_report_2017.pdf. [cited 2018 Oct 23].

[11] World Food Program. The WFP food basket. Rome: World Food Program; 2017.

[12] Clapp Jennifer. Hunger in the Balance: The New Politics of International Food Aid Ithaca: Cornell University Press; 2012.

[13] Global Burden of Disease Collaborators 2015. Healthcare Access and Quality Index based on mortality from causes amenable to personal health care in 195 countries and territories, 1990–2015: a novel analysis from the Global Burden of Disease Study 2015. Lancet. 2017;390: 231–266. 10.1016/S0140-6736(17)30818-8 28528753 10.1016/S0140-6736(17)30818-8 PMC5528124

[14] Boston Consulting Group. Food-restricted voucher or unrestricted cash? How to best support Syrian refugees in Jordan and Lebanon?—April 2017 Boston: Boston Consulting. Group; 2017.

[15] World Food Program. Lebanon Post-Distribution Monitoring Report. Rome: World Food Program; 2014.

[16] Creti P. The Voucher Programme in the Gaza Strip: Mid-Term Review Jerusalem: World Food Program; 2011.

[17] Hidrobo M, Hoddinott J, Peterman A, Margolies A, Moreira V. Cash, food, or vouchers? Evidence from a randomized experiment in northern Ecuador. J Dev Econ. 2014;107: 144–156. 10.1016/j.jdeveco.2013.11.009

[18] Lentz EC, Passarelli S, Barrett CB. The Timeliness and Cost-Effectiveness of the Local and Regional Procurement of Food Aid. World Dev. 2013;49: 9–18. 10.1016/j.worlddev.2013.01.017

[19] Tschirley DL, del Castillo AM. Local and Regional Food Aid Procurement: An Assessment of Experience in Africa and Elements of Good Donor Practice East Lansing: Michigan State University; 2008.

[20] Thow AM, Priyadarshi S. Aid for Trade: an opportunity to increase fruit and vegetable supply. Bull World Health Org. 2013;91: 57–63. 10.2471/BLT.12.106955 23397351 10.2471/BLT.12.106955 PMC3537247

[21] Schwab B, Margolies A, Hoddinott J. Impact Evaluation of Cash and Food Transfers for the Seasonal Emergency Safety Net in Hajjah and Ibb Governorates: Yemen Endline Report Washington: Int Food Policy Res Inst; 2013.

[22] UN High Commissioner for Refugees. Vulnerability assessment of Syrian refugees in Lebanon Geneva: UN High Commissioner for Refugees; 2016.

[23] World Food Program. Food Assistance for the Food-Insecure Populations in the West Bank and Gaza Strip Rome: World Food Program; 2016.

[24] America Oxfam. Food aid: A critical program, ripe for reform Boston: Oxfam America; 2018.

[25] Caro JJ, Briggs AH, Siebert U, Kuntz KM. Modeling good research practices—overview: a report of the ISPOR-SMDM Modeling Good Research Practices Task Force—1. Value Heal J Int Soc Pharmacoeconomics Outcomes Res. 2012;15: 796–803. 10.1016/j.jval.2012.06.012 22999128 10.1016/j.jval.2012.06.012

[26] United Nations Relief and Works Agency. UNRWA in figures Jerusalem: United Nations Relief and Works Agency; 2017.

[27] Micha R, Shulkin ML, Peñalvo JL, Khatibzadeh S, Singh GM, Rao M, et al Etiologic effects and optimal intakes of foods and nutrients for risk of cardiovascular diseases and diabetes: Systematic reviews and meta-analyses from the Nutrition and Chronic Diseases Expert Group (NutriCoDE). PLoS ONE. 2017;12: e0175149 10.1371/journal.pone.0175149 28448503 10.1371/journal.pone.0175149 PMC5407851

[28] World Food Programme. WFP/Turkish Red Crescent E-Voucher Food Card Program Rome: World Food Programme; 2012.

[29] Jordan Emergency Operations. World Food Programme Beneficiary Targeting for Syrian Refugees in the Community Al-Jubaiha, Rasheed District: World Food Programme; 2015.

[30] Atos Consulting and Technology Services, Public Administration International. Improving Food Security for the People of Gaza: Independent Evaluation London: UK Department for International Development; 2015.

[31] Kennedy G, Ballard T, Dop MC. Guidelines for measuring household and individual dietary diversity Rome: Food and Agriculture Organization of the United Nations; 2011.

[32] Schwingshackl L, Missbach B, König J, Hoffmann G. Adherence to a Mediterranean diet and risk of diabetes: a systematic review and meta-analysis. Public Health Nutr. 2015;18: 1292–1299. 10.1017/S1368980014001542 25145972 10.1017/S1368980014001542 PMC10273006

[33] Salas-Salvadó J, Bulló M, Babio N, Martínez-González MÁ, Ibarrola-Jurado N, Basora J, et al Reduction in the Incidence of Type 2 Diabetes With the Mediterranean Diet. Diabetes Care. 2011;34: 14–19. 10.2337/dc10-1288 20929998 10.2337/dc10-1288 PMC3005482

[34] Sarrafzadegan N, Kelishadi R, Sadri G, Malekafzali H, Pourmoghaddas M, Heidari K, et al Outcomes of a comprehensive healthy lifestyle program on cardiometabolic risk factors in a developing country: the Isfahan Healthy Heart Program. Arch Iran Med. 2013;16: 4–11. doi: 013161/AIM.004 23273227

[35] Harati H, Hadaegh F, Momenan AA, Ghanei L, Bozorgmanesh MR, Ghanbarian A, et al Reduction in incidence of type 2 diabetes by lifestyle intervention in a middle eastern community. Am J Prev Med. 2010;38: 628—636.e1. 10.1016/j.amepre.2010.03.003 20494239 10.1016/j.amepre.2010.03.003

[36] Food and Agricultural Organization. International Network of Food Data Systems Rome: Food and Agricultural Organization; 2017.

[37] US Department of Agriculture. USDA National Nutrient Database for Standard Reference Beltville: US Department of Agriculture; 2016.

[38] Hall KD, Sacks G, Chandramohan D, Chow CC, Wang YC, Gortmaker SL, et al Quantification of the effect of energy imbalance on bodyweight. Lancet. 2011;378: 826–837. 10.1016/S0140-6736(11)60812-X 21872751 10.1016/S0140-6736(11)60812-X PMC3880593

[39] Hall KD, Jordan PN. Modeling weight-loss maintenance to help prevent body weight regain. Am J Clin Nutr. 2008;88: 1495–1503. 10.3945/ajcn.2008.26333 19064508 10.3945/ajcn.2008.26333

[40] Hall KD, Butte NF, Swinburn BA, Chow CC. Dynamics of childhood growth and obesity: development and validation of a quantitative mathematical model. Lancet Diabetes Endocrinol. 10.1016/S2213-8587(13)70051-2 10.1016/s2213-8587(13)70051-2 PMC3857695 24349967

[41] Mente A, O’Donnell MJ, Rangarajan S, McQueen MJ, Poirier P, Wielgosz A, et al Association of Urinary Sodium and Potassium Excretion with Blood Pressure. N Engl J Med. 2014;371: 601–611. 10.1056/NEJMoa1311989 25119606 10.1056/NEJMoa1311989

[42] Mensink RP, Zock PL, Kester ADM, Katan MB. Effects of dietary fatty acids and carbohydrates on the ratio of serum total to {HDL} cholesterol and on serum lipids and apolipoproteins: a meta-analysis of 60 controlled trials. Am J Clin Nutr. 2003;77: 1146–1155. Available from: http://ajcn.nutrition.org/content/77/5/1146. 10.1093/ajcn/77.5.1146 12716665 10.1093/ajcn/77.5.1146

[43] Narayan KM V., Boyle JP, Thompson TJ, Gregg EW, Williamson DF. Effect of BMI on lifetime risk for diabetes in the U.S. Diabetes Care. 2007;30: 1562–1566. 10.2337/dc06-2544 17372155 10.2337/dc06-2544

[44] Ajala O, English P, Pinkney J. Systematic review and meta-analysis of different dietary approaches to the management of type 2 diabetes. Am J Clin Nutr. 2013;97: 505–516. 10.3945/ajcn.112.042457 23364002 10.3945/ajcn.112.042457

[45] Robert CP, Casella G. Introducing Monte Carlo Methods with R New York: Springer Verlag; 2009.

[46] Sanders GD, Neumann PJ, Basu A, Brock DW, Feeny D, Krahn M, et al Recommendations for Conduct, Methodological Practices, and Reporting of Cost-effectiveness Analyses: Second Panel on Cost-Effectiveness in Health and Medicine. JAMA. 2016;316: 1093–1103. 10.1001/jama.2016.12195 27623463 10.1001/jama.2016.12195

[47] United Nations. World Population Prospects Geneva: United Nations; 2017.

[48] Whelton PK, Carey RM, Aronow WS, Casey DE, Collins KJ, Himmelfarb CD, et al 2017 ACC/AHA/AAPA/ABC/ACPM/AGS/APhA/ASH/ASPC/NMA/PCNA Guideline for the Prevention, Detection, Evaluation, and Management of High Blood Pressure in Adults: A Report of the American College of Cardiology/American Heart Association Task Force on Clinical Pr. J Am Coll Cardiol. 2017; 24430 10.1016/j.jacc.2017.11.006

[49] American Diabetes Association. 2. Classification and Diagnosis of Diabetes: Standards of Medical Care in Diabetes-2018. Diabetes Care. 2018;41: S13–S27. 10.2337/dc18-S002 29222373 10.2337/dc18-S002

[50] Hajifathalian K, Ueda P, Lu Y, Woodward M, Ahmadvand A, Aguilar-Salinas CA, et al A novel risk score to predict cardiovascular disease risk in national populations (Globorisk): a pooled analysis of prospective cohorts and health examination surveys. Lancet Diabetes Endocrinol. 2015;3: 339–355. 10.1016/S2213-8587(15)00081-9 25819778 10.1016/S2213-8587(15)00081-9 PMC7615120

[51] Ueda P, Woodward M, Lu Y, Hajifathalian K, Al-Wotayan R, Aguilar-Salinas CA, et al Laboratory-based and office-based risk scores and charts to predict 10-year risk of cardiovascular disease in 182 countries: a pooled analysis of prospective cohorts and health surveys. Lancet Diabetes Endocrinol. 2017;5: 196–213. 10.1016/S2213-8587(17)30015-3 28126460 10.1016/S2213-8587(17)30015-3 PMC5354360

[52] Basu S, Sussman JB, Berkowitz SA, Hayward RA, Yudkin JS. Development and validation of Risk Equations for Complications Of type 2 Diabetes (RECODe) using individual participant data from randomised trials. Lancet Diabetes Endocrinol. 2017;5: 788–798. 10.1016/S2213-8587(17)30221-8 28803840 10.1016/S2213-8587(17)30221-8 PMC5769867

[53] Basu S, Sussman J, Berkowitz SA, Hayward RA, Bertoni AG, Correa A, et al Validation of Risk Equations for Complications of Type 2 Diabetes (RECODe) Using Individual Participant Data From Diverse Longitudinal Cohorts in the U.S. Diabetes Care. 2018;41: 586–95. 10.2337/dc17-2002 29269511 10.2337/dc17-2002 PMC5829967

[54] Melito T. International Food Assistance: Local and Regional Procurement Can Enhance the Efficiency of U.S. Food Aid, But Challenges May Constrain Its Implementation Washington: US Government Accountability Office; 2009.

[55] Palestinian Central Bureau of Statistics. Palestine—Expenditure and Consumption Survey Jerusalem: Palestinian Central Bureau of Statistics; 2011.

[56] Marks GC, Hughes MC, van der Pols JC. Relative Validity of Food Intake Estimates Using a Food Frequency Questionnaire Is Associated with Sex, Age, and Other Personal Characteristics. J Nutr. Oxford University Press; 2006;136: 459–465. 10.1093/jn/136.2.459 16424128 10.1093/jn/136.2.459

[57] Rosato V, Temple NJ, La Vecchia C, Castellan G, Tavani A, Guercio V. Mediterranean diet and cardiovascular disease: a systematic review and meta-analysis of observational studies. Eur J Nutr. Springer Berlin Heidelberg; 2017; 1–19. 10.1007/s00394-017-1582-0 29177567 10.1007/s00394-017-1582-0

[58] Murray CJL, Barber RM, Foreman KJ, Abbasoglu Ozgoren A, Abd-Allah F, et al Global, regional, and national disability-adjusted life years (DALYs) for 306 diseases and injuries and healthy life expectancy (HALE) for 188 countries, 1990–2013: quantifying the epidemiological transition. Lancet. 2015;386: 2145–2191. 10.1016/S0140-6736(15)61340-X 26321261 10.1016/S0140-6736(15)61340-X PMC4673910

[59] Basu S. Modeling Public Health and Healthcare Systems Oxford; New York: Oxford University Press; 2017.

[60] Venables WN, Ripley BD, Venables WN. Modern applied statistics with S New York: Springer; 2003.

[61] Wickham H, Grolemund G. R for data science: import, tidy, transform, visualize, and model data. Available from: http://r4ds.had.co.nz/. [cited 2018 Oct 23].

[62] Choi SE, Brandeau ML, Basu S. Expansion of the National Salt Reduction Initiative: A Mathematical Model of Benefits and Risks of Population-Level Sodium Reduction. Med Decis Mak An Int J Soc Med Decis Mak. 2016;36: 72–85. 10.1177/0272989X15583846 25926284 10.1177/0272989X15583846 PMC4626435

[63] Aune D, Keum N, Giovannucci E, Fadnes LT, Boffetta P, Greenwood DC, et al Whole grain consumption and risk of cardiovascular disease, cancer, and all cause and cause specific mortality: systematic review and dose-response meta-analysis of prospective studies. BMJ. 2016;353: i2716 10.1136/bmj.i2716 27301975 10.1136/bmj.i2716 PMC4908315

[64] Friedmann H. Distance and durability: Shaky foundations of the world food economy. Third World Q. 1992;13: 371–383. 10.1080/01436599208420282

[65] Van Wassenhove LN. Humanitarian aid logistics: supply chain management in high gear. J Oper Res Soc. 2006;57: 475–489. 10.1057/palgrave.jors.2602125

[66] Pettit S, Beresford A. Critical success factors in the context of humanitarian aid supply chains. Int J Phys Distrib Logist Manag. 2009;39: 450–468. 10.1108/09600030910985811

[67] Oloruntoba R, Gray R. Humanitarian aid: an agile supply chain? Supply Chain Manag An Int J. 2006;11: 115–120. 10.1108/13598540610652492

