# Supplementary material for: Reducing chronic disease through changes in food aid: A microsimulation of nutrition and cardiometabolic disease among Palestinian refugees in the Middle East
Source: PLoS Med. 2018 Nov 20;15(11):e1002700. doi: 10.1371/journal.pmed.1002700 (PMC6245519; doi:10.1371/journal.pmed.1002700)
Supplement: S1 Text — (PDF) [file pmed.1002700.s001.pdf]

S1 Text

1 Prospective analysis plan

The analysis will proceed in three stages. In stage 1, the demographic population of the 5.3 million registered refugees in the Middle East, per United Nations definitions [1], will be simulated using a microsimulation approach that captures both the marginal distributions of key demographic risk factors for chronic disease (age, sex, etc.) and the corresponding nutritional intake features (kcals/person/day in each of several food categories based on food frequency questionnaire) and health attributes (sampled from the UNRWA electronic health record). The proportion of refugees receiving food aid will be simulated, and the total nutritional intake will be simulated, accounting for both aid- and non-aid-based food consumption.

In stage 2, the health outcomes associated with current nutritional intake patterns will be estimated by simulating the incidence of hypertension, type 2 diabetes mellitus, and associated atherosclerotic cardiovascular disease events and microvascular disease events using locally-recalibrated versions of the Globorisk and RECODe equations, respectively [2,3].

In stage 3, the changes in health outcomes associated with changes in nutritional intake from conversion of traditional in-kind food aid to electronic (e-voucher) debit card aid restricted to food purchases will be simulated by resampling from the distributions of food intake by food category among refugees in field-based pilot studies of the transition [4,5]. The comparative effectiveness and cost-effectiveness of the food aid change will be estimated over the full life-course of the population was simulated for any persons alive during a 10-year policy planning horizon (2016-2025), with a standard 3% annual discount rate (**S4 Table**).

Sensitivity analyses will include an alternative conversion of in-kind food aid to cash, and a threshold analysis of the amount of grain that must be converted to fruits and vegetables under a fixed aid budget to achieve significant reduction in each cardiovascular disease and microvascular disease outcome variable. Uncertainty analyses

will involve repeated Monte Carlo sampling with replacement from all input parameter distributions.

2 Model equations

An *in vivo* model of energy metabolism was used to estimate how much body weight will change for each individual given a change in calorie intake, and over what time frame. The model was previously validated against several weight loss experiments [6–8]. Equation 1 estimates how much an individual's body weight *M(t)* changes after a change in calorie consumption λ at time *t*:

(1) $$\frac{dM(t)}{dt} = [\lambda(t) - \varepsilon(t)(M(t) - M_0)] / \rho \,.$$

In equation (1), M_0 is the initial body weight prior to the calorie consumption change, ρ is the weight change associated with the energy density (the conversion of new kilocalories into kilograms), and ε captures energy expenditure (accounting for how much of the intake is expended through physical activity versus how much remains as new body weight). Because energy intake and expenditure do not produce an instantaneous change in body weight, this equation captures how changes in calories and energy expenditure result in a non-linear change in body weight over time, conditional upon constraints inherent to human metabolism. The following two equations describe the internal physiology of protein, fat and glucose metabolism using the variables defined in **S6 Table**:

(2) $$\rho = \frac{\eta_f + \rho_f + \alpha\eta_l + \alpha\rho_l}{(1-\beta)(1+\alpha)}$$

(3) $$\varepsilon(t) = \frac{1}{(1-\beta)}\left(\frac{\gamma_f + \alpha\gamma_l}{(1+\alpha)} + \delta(t)\right)$$

Equation (2) describes change in energy as calories are metabolized (capturing the efficiency of fat and protein synthesis, and the dynamics of thermogenesis and change in lean mass per change in fat mass, see **S6 Table**), while Equation (3) describes how stored energy is broken down during catabolism (based on resting metabolic rates, **S6 Table**). A full derivation of the equations and their validation against experimental

data with head-to-head comparison against alternative models has been published previously [6,7]. The parameter $\delta(t)$ reflects the calories expended through physical activity as a function of the physical activity level (PAL[9]) and the resting metabolic rate[10] (**S6 Table**). Of note, the equations reduce to the following steady-state prediction for the ultimate change in body weight for a given change in intake or expenditure:

(4) $$\Delta BW = \frac{(1-\beta)\lambda - M_0\Delta\delta}{\delta_{init} + \Delta\delta + \gamma_L - \Phi(\gamma_L - \gamma_F)},$$

where the initial PAL and change in PAL are defined as δ_{init} and $\Delta\delta$, respectively, and Φ is the ratio of the change in fat to fat-free mass.

Alternate equations are validated for persons <18 years of age, to account for growth trajectories [8]. The alternative equations involve estimating the net change in kilograms among children given a change in kilocalories per person per day by Equation 5 for males and Equation 6 for females:

(5) $$\Delta kg = (\Delta\ kcal/person/day)/(68 - 2.5 \times age)$$

(6) $$\Delta kg = (\Delta\ kcal/person/day)/(62 - 2.2 \times age)$$

Changes in sodium and potassium were used to estimate changes in systolic blood pressure using regression estimates from a large, international prospective epidemiological study (N = 102,216) with estimates of 24-hour sodium (Na, g/person/day) and potassium (K, g/person/day) excretion correlated to measured systolic blood pressure (SBP, mmHg) and diastolic blood pressure (DBP, mmHg) [11].

(7) $$\Delta SBP = \beta_1 Na + \beta_2 K$$

(8) $$\Delta DBP = \beta_3 Na + \beta_4 K$$

where β_1 is 1.96 (SE: 0.18), β_2 is -2.60 (SE: 0.46), β_3 is 0.83 (SE: 0.10), and β_4 is -0.96 (SE: 0.25).

Change in fatty acids were used to estimate changes in total (Totchol, mmol/L) and high-density lipoprotein cholesterol (HDL, mmol/L) using regression estimates from a meta-analysis of 60 trials (N = 1,672) [12].

(9) $$\Delta Totchol = \beta_5 \Delta SFA + \beta_6 \Delta MUFA + \beta_7 \Delta PUFA$$

(10) $$\Delta HDL = \beta_8 \Delta SFA + \beta_9 \Delta MUFA + \beta_{10} \Delta PUFA$$

where β_5 is 0.036 (SE: 0.004), β_6 is -0.006 (SE: 0.003), β_7 is -0.021 (SE: 0.003), β_8 is 0.010 (SE: 0.002), β_9 is 0.008 (SE: 0.002), and β_{10} is 0.006 (SE: 0.002).

Change in the Mediterranean Diet Score (MDS) was computed based on expressing the kilocalories of intake in terms of grams per person per day and computing the MDS using the gram-based tabular scoring tool published previously by the National Audit of Cardiac Rehabilitation [13].

Change body mass index (BMI, kg/m^2) and in MDS (scale from 0 to 14) were used to estimate the ratio of new to baseline hemoglobin A1c (RHbA1c) based on regression estimates from meta-analyses of 20 prospective studies and risk projections (N=3,073) [14–16].

(11) $RHbA1c = (1 + \beta_{11}\Delta BMI) \times (1 + \beta_{12}\Delta MDS)$

where β_{11} is 0.025 (SE: 0.003), β_{12} is -0.035 (SE: 0.005).

The probability of cardiovascular disease events (P(CVD) for individual i of age j in location k from time t to t') was estimated by the Globorisk equations validated among eight prospective cohort studies including populations in the Middle East [2,17].

(12) $P_i(CVD) = 1 - \prod_{j=t}^{t+t'}(\exp\,(-\varphi_i(j))$

(13) $\varphi_i(j) = \varphi_{0,k}(t)\exp\,\left[\sum_{l=1}^{4}\beta_l(X_{i,l} - \bar{X}_{l,k,t}) + \sum_{l=1}^{4}\delta_l t(X_{i,l} - \bar{X}_{l,k,t}) + \sum_{l=3}^{4}\gamma_l sex_i(X_{i,l} - \bar{X}_{l,k,t})\right]$

where $\varphi_i(j)$ is the CVD hazard function, $\varphi_{0,k}(t)$ is the average age t and sex-specific CVD rate in location k (estimated by the Global Burden of Disease Study and listed at globorisk.org), $X_{i,l}$ is the value of each risk factor l for individual i in location l, $\bar{X}_{l,k,t}$ is the mean location-specific value of risk factor l at age t in country k. The risk factors with main effects β_l are SBP, total cholesterol, diabetes status, and tobacco smoking; all four risk factors have additional age interaction term coefficients δ_l, while the latter two have additional sex interaction term coefficients γ_l. Coefficients are reproduced in **S7 Table**.

The probabilities of microvascular events and all-cause mortality were estimated by RECODe equations in which the baseline hazard of each event was recalibrated such that the baseline prevalence of microvascular events and baseline death rate matched

the UNRWA and partner health audit data (**Table 1**) [3,18]. The RECODe equations estimate the probability of end-stage renal disease, diabetic retinopathy, diabetic neuropathy, and all-cause mortality as per equation (14).

$$(14) \quad P(event) = 1 - \lambda^{\exp\left(\beta_0 + \sum_{l=1}^{12} \beta_l X_i\right)}$$

where λ is the baseline hazard of each event, X_i is the value of each risk factor l for individual i, β_0 is an intercept term, and β_l are the effect coefficients corresponding to each of the risk factors of age, sex, SBP, blood pressure treatment, CVD history, oral diabetes treatment, serum creatinine, total cholesterol, HDL cholesterol, hemoglobin A1c, and urine microalbumin/creatinine ratio. Coefficients are reproduced in **S8 Table**.

3 Cost-effectiveness analysis

The impact inventory for the cost-effectiveness analysis is provided in **S4 Table**, and a CHEERS checklist in **S9 Table.**

4 References

1. United Nations Relief and Works Agency. UNRWA in figures. Jerusalem: UNRWA; 2017.
2. Hajifathalian K, Ueda P, Lu Y, Woodward M, Ahmadvand A, Aguilar-Salinas CA, et al. A novel risk score to predict cardiovascular disease risk in national populations (Globorisk): a pooled analysis of prospective cohorts and health examination surveys. Lancet Diabetes Endocrinol. 2015 May;3(5):339–55.
3. Basu S, Sussman JB, Berkowitz SA, Hayward RA, Yudkin JS. Development and validation of Risk Equations for Complications Of type 2 Diabetes (RECODe) using individual participant data from randomised trials. Lancet Diabetes Endocrinol [Internet]. 2017 Oct 1 [cited 2018 Mar 8];5(10):788–98. Available from: https://www.sciencedirect.com/science/article/pii/S2213858717302218
4. Creti P. The Voucher Programme in the Gaza Strip: Mid-Term Review. World Food Program Jerusalem. 2011;
5. World Food Program. Lebanon Post-Distribution Monitoring Report. Rome: WFP; 2014.
6. Hall KD, Sacks G, Chandramohan D, Chow CC, Wang YC, Gortmaker SL, et al. Quantification of the effect of energy imbalance on bodyweight. Lancet. 2011;378(9793):826–37.
7. Hall KD, Jordan PN. Modeling weight-loss maintenance to help prevent body weight regain. Am J Clin Nutr. 2008 Dec;88(6):1495–503.
8. Hall KD, Butte NF, Swinburn BA, Chow CC. Dynamics of childhood growth and obesity: development and validation of a quantitative mathematical model. Lancet Diabetes Endocrinol.
9. Heymsfield SB, Harp JB, Rowell PN, Nguyen AM, Pietrobelli A. How much may I eat? Calorie estimates based upon energy expenditure prediction equations. Obes Rev an Off J Int Assoc Study Obes. 2006 Nov;7(4):361–70.
10. Mifflin MD, St Jeor ST, Hill LA, Scott BJ, Daugherty SA, Koh YO. A new predictive equation for resting energy expenditure in healthy individuals. Am J Clin Nutr. 1990 Feb;51(2):241–7.

11. Mente A, O'Donnell MJ, Rangarajan S, McQueen MJ, Poirier P, Wielgosz A, et al. Association of Urinary Sodium and Potassium Excretion with Blood Pressure. N Engl J Med. 2014 Aug;371(7):601–11.
12. Mensink RP, Zock PL, Kester ADM, Katan MB. Effects of dietary fatty acids and carbohydrates on the ratio of serum total to {HDL} cholesterol and on serum lipids and apolipoproteins: a meta-analysis of 60 controlled trials. Am J Clin Nutr [Internet]. 2003 May [cited 2017 Dec 18];77(5):1146–55. Available from: http://ajcn.nutrition.org/content/77/5/1146
13. Rehabilitation NA of C. The Mediterranean Diet Score Tool. London: NACR; 2013.
14. Schwingshackl L, Missbach B, König J, Hoffmann G. Adherence to a Mediterranean diet and risk of diabetes: a systematic review and meta-analysis. Public Health Nutr. 2015 May;18(7):1292–9.
15. Narayan KM V., Boyle JP, Thompson TJ, Gregg EW, Williamson DF. Effect of BMI on lifetime risk for diabetes in the U.S. Diabetes Care. 2007 Jun;30(6):1562–6.
16. Salas-Salvadó J, Bulló M, Babio N, Martínez-González MÁ, Ibarrola-Jurado N, Basora J, et al. Reduction in the Incidence of Type 2 Diabetes With the Mediterranean Diet. Diabetes Care [Internet]. 2011;34(1):14–9. Available from: https://www.ncbi.nlm.nih.gov/pmc/articles/PMC3005482/
17. Ueda P, Woodward M, Lu Y, Hajifathalian K, Al-Wotayan R, Aguilar-Salinas CA, et al. Laboratory-based and office-based risk scores and charts to predict 10-year risk of cardiovascular disease in 182 countries: a pooled analysis of prospective cohorts and health surveys. Lancet Diabetes Endocrinol. 2017 Mar;5(3):196–213.
18. Basu S, Sussman JB, Berkowitz SA, Hayward RA, Bertoni AG, Correa A, et al. Validation of Risk Equations for Complications of Type 2 Diabetes (RECODe) Using Individual Participant Data From Diverse Longitudinal Cohorts in the U.S. Diabetes Care. 2017 Dec;dc172002.
