# Supplementary material for: Reducing chronic disease through changes in food aid: A microsimulation of nutrition and cardiometabolic disease among Palestinian refugees in the Middle East
Source: PLoS Med. 2018 Nov 20;15(11):e1002700. doi: 10.1371/journal.pmed.1002700 (PMC6245519; doi:10.1371/journal.pmed.1002700)
Supplement: S1 Table — (DOCX) [file pmed.1002700.s002.docx]

S1 Table: Estimated distribution of kilocalories consumed per person per day by food type, under traditional food parcel delivery versus electronic food aid delivery.

| **Foods** | **Traditional food parcel delivery (“in kind” food aid)** | **Electronic debit card delivery (“e-voucher”)** |  |
| --- | --- | --- | --- |
|  | **Kcals/person/day, Mean (SD)** | **Kcals/person/day, Mean (SD)** |  |
| **Bread and cereals** |  |  |  |
| Short-grain rice | 150.46 (51.42) | 159.28 (53.49) |  |
| Long-grain rice | 19.21 (11.66) | 19.3 (10.99) |  |
| Wheat | 20.39 (2.59) | 21.98 (2.66) |  |
| Local wheat flour | 22.4 (1.86) | 22.73 (2.01) |  |
| Imported white flour | 414.1 (154.94) | 417.75 (149.11) |  |
| Different kinds of bread | 270.95 (85.95) | 245.75 (91.17) |  |
| Qurshallah (twice baked sesame bread) | 10.17 (3.17) | 10.32 (3.39) |  |
| Cookies stuffed with dates | 0.9 (0.18) | 0.84 (0.19) |  |
| Sesame bar | 0.09 (0.05) | 0.09 (0.05) |  |
| Macaroni | 6.74 (2.72) | 6.43 (2.96) |  |
| Noodles | 6.92 (2.56) | 6.44 (2.74) |  |
| Stuffed biscuit | 22.2 (8.23) | 22.48 (7.42) |  |
| Salted biscuit or local | 3.58 (0.32) | 3.62 (0.3) |  |
| Semolina | 8 (1.05) | 8.21 (1.13) |  |
| Crushed wheat | 1.51 (0.59) | 1.57 (0.62) |  |
| Roasted green wheat | 3.37 (2.3) | 3.2 (2.4) |  |
| Starch | 1.24 (0.38) | 1.26 (0.42) |  |
| Cake & cookies | 3.27 (0.73) | 3.36 (0.75) |  |
| Infants products (Cerelac, farleys) | 0.63 (0.17) | 0.59 (0.17) |  |
| Breakfast cereals (e.g., cornflakes) | 0.24 (0.13) | 0.22 (0.13) |  |
| Corn chips, popcorn | 4.9 (1.11) | 4.69 (1.15) |  |
| Oriental deserts (Kunafa, etc.) | 18.5 (3.25) | 18.38 (2.96) |  |
| Ready made maftool (couscous) | 1.18 (0.16) | 1.21 (0.16) |  |
| Dough pastry | 4.53 (1.84) | 4.92 (1.82) |  |
| Other | 3.46 (2.06) | 3.79 (2.24) |  |
| **Meat & poultry** |  |  |  |
| Fresh goats & sheep meat | 22.64 (9.71) | 29.67 (14.12) |  |
| Frozen goats & sheep meat | 0.11 (0.06) | 0.15 (0.08) |  |
| Fresh bovine meat | 29.2 (4.32) | 42.58 (5.61) |  |
| Frozen cow meat | 9.78 (3.92) | 13.8 (5.71) |  |
| Fresh camel meat | 0.04 (0.02) | 0.05 (0.03) |  |
| Fresh or frozen rabbit meat | 0.46 (0.46) | 0.64 (0.67) |  |
| Slaughtered poultry | 25.73 (17.19) | 32.8 (23.61) |  |
| Frozen chicken | 5.02 (2.76) | 6.57 (3.57) |  |
| Fresh turkey | 5.05 (0.64) | 6.75 (0.87) |  |
| Squab fresh or frozen | 0.82 (0.67) | 1.16 (0.99) |  |
| Other fresh or frozen birds | 0.43 (0.53) | 0.58 (0.7) |  |
| Processed lamb/ beef (hamburger, mortadella) | 0.45 (0.22) | 0.63 (0.32) |  |
| Tinned meat | 0.7 (0.59) | 0.93 (0.78) |  |
| Fresh chicken and turkey liver | 1.33 (0.28) | 1.7 (0.41) |  |
| Live poultry (purchased before butchering) | 124.82 (18.87) | 174.94 (23.99) |  |
| Frozen turkey | 4.33 (1.12) | 5.6 (1.49) |  |
| Processed poultry meat (mortadella, sausages) | 9.16 (1.95) | 11.27 (2.7) |  |
| Fresh lamb liver | 0.57 (0.38) | 0.75 (0.5) |  |
| Fresh cow liver | 0.64 (0.01) | 0.81 (0.01) |  |
| Inside organs and limbs of slaughtered animal | 1.94 (0.53) | 2.67 (0.74) |  |
| Other meats | 3.88 (2.35) | 5.42 (2.91) |  |
| **Fish & sea product** |  |  |  |
| Fish fresh | 6.42 (6.4) | 8.33 (9.46) |  |
| Fish frozen | 11.35 (0.13) | 13.93 (0.2) |  |
| Fish salted | 0.66 (0.56) | 0.93 (0.69) |  |
| Smoked fish | 0.06 (0.07) | 0.08 (0.1) |  |
| Tinned, sardines | 0.72 (0.09) | 0.94 (0.12) |  |
| Tuna, tinned | 1.01 (0.56) | 1.26 (0.76) |  |
| Other | 0.07 (0.09) | 0.1 (0.11) |  |
| **Dairy and eggs** |  |  |  |
| Fresh or pasteurized milk | 44.82 (14.13) | 130.56 (44.35) |  |
| Condensed liquid milk | 0.7 (0.64) | 2.01 (1.82) |  |
| Powder milk | 4.82 (2.94) | 13.03 (8.41) |  |
| Infants powder milk | 2.06 (0.48) | 5.35 (1.44) |  |
| Tinned yogurt | 49.11 (16.49) | 137.68 (45.93) |  |
| Yogurt, liquid | 3.1 (0.18) | 9.64 (0.55) |  |
| Tinned yogurt paste (labaneyh) | 5.91 (3.79) | 18.39 (10.21) |  |
| Soft white cheese | 10.37 (2.86) | 27.24 (7.87) |  |
| Home made cheese | 1.78 (1.23) | 5.59 (3.39) |  |
| Cooked cheese for sandwich | 0.87 (0.27) | 2.56 (0.84) |  |
| Processed cheese | 2.49 (0.53) | 6.57 (1.64) |  |
| Cream | 0.04 (0.01) | 0.13 (0.03) |  |
| Yogurt in solid form (Jamid) or (Kishik) | 1.87 (1.23) | 5.32 (3.69) |  |
| Other dairy products | 0.89 (0.33) | 2.57 (0.97) |  |
| Eggs | 39.66 (2.66) | 104.06 (7.38) |  |
| Other canned cheese | 0.93 (1.17) | 2.6 (3.08) |  |
| **Oils and fats** |  |  |  |
| Olive oil | 59.93 (30.27) | 58.29 (28.67) |  |
| Corn oil | 66.9 (26.21) | 58.9 (23.97) |  |
| Sunflower oil | 16.85 (17.03) | 16.88 (15.14) |  |
| Soya oil | 22.7 (25.48) | 20.57 (23.64) |  |
| Palm Kernel oil | 0.21 (0.23) | 0.21 (0.24) |  |
| Vegetable fat | 7.45 (0.51) | 6.79 (0.47) |  |
| Animal fat | 0.24 (0.15) | 0.21 (0.14) |  |
| Margarine/ butter | 2.51 (1.11) | 2.47 (1.1) |  |
| Other oils & fats | 0.33 (0.23) | 0.29 (0.22) |  |
| **Fresh fruits** |  |  |  |
| Orange | 16.64 (2.26) | 22.54 (3.01) |  |
| Mandarin | 5.02 (1.05) | 5.93 (1.41) |  |
| Pomelos | 1.07 (0.64) | 1.51 (0.77) |  |
| Grapefruit | 0.57 (0.54) | 0.74 (0.66) |  |
| Lemon | 7.39 (0.93) | 9.33 (1.15) |  |
| Bananas | 15.98 (3.11) | 22.15 (3.87) |  |
| Apples | 21.05 (1.67) | 28.92 (2.04) |  |
| Grapes | 6.42 (0.9) | 8.48 (1.18) |  |
| Water melon | 17.09 (2.62) | 21.45 (3.36) |  |
| Melon | 3.55 (0.97) | 4.13 (1.29) |  |
| Apricots | 1.45 (0.58) | 1.96 (0.75) |  |
| Plums | 2.16 (0.9) | 2.53 (1.06) |  |
| Cherries | 0.08 (0.05) | 0.11 (0.07) |  |
| Peaches | 5.78 (0.24) | 7.71 (0.29) |  |
| Strawberries | 1.51 (0.71) | 2.08 (1) |  |
| Pears | 3.04 (0.51) | 4.15 (0.71) |  |
| Guava | 1.8 (0.44) | 2.38 (0.51) |  |
| Pomegranate | 0.31 (0.08) | 0.37 (0.11) |  |
| Figs | 0.6 (0.09) | 0.74 (0.11) |  |
| Prickly pear | 0.75 (0.72) | 1 (1.02) |  |
| Khakis | 1.15 (0.08) | 1.53 (0.09) |  |
| Dates | 0.68 (0.54) | 0.83 (0.65) |  |
| Indian apricot | 0.36 (0.02) | 0.44 (0.03) |  |
| Mango | 2.82 (1.84) | 3.81 (2.42) |  |
| Almonds, green | 0.1 (0.04) | 0.13 (0.05) |  |
| Other fresh fruits | 4.38 (0.28) | 6.18 (0.33) |  |
| **Tinned fruits** |  |  |  |
| Tinned pine apple | 0.02 (0.01) | 0.03 (0.02) |  |
| Other tinned fruits | 0.01 (0.01) | 0.01 (0.01) |  |
| **Dried fruits** |  |  |  |
| Dried figs | 0.01 (0) | 0.02 (0.01) |  |
| Dried grapes | 0.04 (0.01) | 0.06 (0.02) |  |
| Dried dates | 1.37 (0.31) | 1.9 (0.44) |  |
| Other dried fruits | 0.03 (0.02) | 0.04 (0.03) |  |
| **Nuts** |  |  |  |
| Pistachio | 0.19 (0.17) | 0.12 (0.1) |  |
| Peanuts | 1.5 (1.16) | 0.9 (0.63) |  |
| Almond | 0.5 (0.13) | 0.31 (0.07) |  |
| Hazelnuts | 0.27 (0.14) | 0.17 (0.08) |  |
| Water melon seeds | 1.61 (0.69) | 1.01 (0.36) |  |
| Pumpkin seeds | 0.12 (0.01) | 0.07 (0) |  |
| Sun flower seeds | 1.25 (0.35) | 0.69 (0.21) |  |
| Walnut | 0.04 (0.02) | 0.03 (0.01) |  |
| Assorted nuts | 2.33 (1.02) | 1.44 (0.59) |  |
| Other nuts | 0.42 (0.33) | 0.23 (0.17) |  |
| **Fresh vegetables** |  |  |  |
| Tomatoes | 58.6 (9.17) | 66.92 (9.56) |  |
| Cucumber | 25.86 (3.73) | 31.41 (3.89) |  |
| Egyptian cucumber | 1.08 (0.06) | 1.38 (0.06) |  |
| Carrot | 4.2 (0.35) | 5.13 (0.39) |  |
| Eggplant | 13.52 (3.2) | 15.32 (3.73) |  |
| Marrow | 11.8 (1.57) | 13.72 (1.92) |  |
| Pumpkin | 0.45 (0.25) | 0.56 (0.28) |  |
| Gourd | 0.25 (0.24) | 0.27 (0.29) |  |
| Green beans | 1.75 (0.65) | 1.95 (0.81) |  |
| Green okra | 1.37 (0.24) | 1.62 (0.29) |  |
| Green broad beans | 1.48 (0.39) | 1.82 (0.46) |  |
| Green Jews mallow (spinach soup) | 8.23 (0.98) | 9.89 (1.22) |  |
| Green peppers | 4.1 (0.94) | 4.8 (1.05) |  |
| Spinach | 2.91 (0.5) | 3.59 (0.58) |  |
| Cauliflower | 12.65 (5.27) | 14.85 (5.92) |  |
| Cabbage | 6.73 (0.23) | 8.11 (0.24) |  |
| Green cow peas | 0.54 (0.32) | 0.63 (0.36) |  |
| Green pea | 1.64 (1.2) | 1.72 (1.39) |  |
| Lettuce | 0.95 (0.12) | 1.07 (0.15) |  |
| Grapes leaves | 0.63 (0.15) | 0.71 (0.18) |  |
| Turnip | 1.28 (0.51) | 1.56 (0.54) |  |
| Yellow corn | 2 (0.42) | 2.38 (0.52) |  |
| Green thyme | 0.51 (0.36) | 0.6 (0.41) |  |
| Green sage | 0.1 (0.06) | 0.11 (0.07) |  |
| Parsley | 1.05 (0.6) | 1.2 (0.69) |  |
| Mint | 0.19 (0.03) | 0.2 (0.04) |  |
| Fennel | 0.4 (0.29) | 0.44 (0.31) |  |
| Radish | 0.95 (0.11) | 1.19 (0.12) |  |
| Spring onions | 0.55 (0.19) | 0.69 (0.22) |  |
| Green olives (not pickled) | 0.65 (0.62) | 0.75 (0.75) |  |
| Other | 10.61 (0.61) | 11.46 (0.68) |  |
| **Frozen vegetables** |  |  |  |
| Frozen green peas | 0.91 (0.33) | 1.15 (0.41) |  |
| Frozen mixed vegetables | 0.18 (0.04) | 0.19 (0.05) |  |
| Frozen green okra | 0.12 (0.1) | 0.14 (0.12) |  |
| Pease and carrots | 0.17 (0.09) | 0.19 (0.11) |  |
| Other frozen vegetables | 0.11 (0.06) | 0.12 (0.06) |  |
| **Legumes & Vegetables Dried or Tinned** |  |  |  |
| Lentils | 5.72 (2.94) | 3.32 (1.67) |  |
| Crushed lentils | 3.11 (0.56) | 1.78 (0.34) |  |
| Dry chick beans | 3.95 (1.39) | 2.35 (0.87) |  |
| Dry fava beans | 1.44 (1.32) | 0.91 (0.8) |  |
| Dry beans | 1.41 (0.28) | 0.75 (0.16) |  |
| Dry yellow corn | 0.63 (0.11) | 0.38 (0.07) |  |
| Lupine | 0.48 (0.11) | 0.29 (0.07) |  |
| Other legumes and vegetables dried | 0.63 (0.08) | 0.35 (0.05) |  |
| Broad beans (tinned) | 0.72 (0.09) | 0.41 (0.05) |  |
| Chick beans, tinned or crushed | 3.25 (0.4) | 1.91 (0.25) |  |
| Dried & tinned sweet beans | 0.32 (0.01) | 0.19 (0.01) |  |
| Tinned sweet beans | 0.18 (0.11) | 0.1 (0.06) |  |
| Onions | 35.67 (11.69) | 20.87 (6.35) |  |
| Garlic | 2.01 (0.26) | 1.11 (0.16) |  |
| Tomato paste or solid (tinned) | 6.88 (0.43) | 4.15 (0.23) |  |
| Other tinned vegetables | 0.42 (0.17) | 0.26 (0.1) |  |
| **Tubers** |  |  |  |
| Potato | 98.31 (7.73) | 55.63 (4.66) |  |
| Sweet potato | 1.44 (1.26) | 0.83 (0.66) |  |
| Potato slices (frozen or tinned) | 0.02 (0.01) | 0.01 (0.01) |  |
| **Sugar and Confectionery** |  |  |  |
| Sugar | 45.64 (2.22) | 40.77 (2.07) |  |
| Halawa (sweet confectionary) | 0.94 (0.02) | 0.88 (0.02) |  |
| Treacle (uncrystallised syrup) | 0.07 (0.01) | 0.06 (0.01) |  |
| Jam | 0.38 (0.08) | 0.37 (0.07) |  |
| Turkish delight (gel of starch and sugar) | 0.3 (0.02) | 0.27 (0.02) |  |
| Honey | 0.27 (0.03) | 0.24 (0.03) |  |
| Local chocolate | 0.78 (0.58) | 0.7 (0.57) |  |
| Imported chocolate | 4.35 (0.77) | 3.91 (0.73) |  |
| Sweet | 0.59 (0.31) | 0.51 (0.31) |  |
| Toffee | 0.29 (0.09) | 0.28 (0.08) |  |
| Chewing gum | 0.2 (0.01) | 0.19 (0.01) |  |
| Bonbon, citrus products, | 0.86 (0.19) | 0.72 (0.16) |  |
| Qamar deen (made of apricot), malban | 0.1 (0.02) | 0.09 (0.02) |  |
| Ice-cream | 3.9 (0.42) | 3.38 (0.37) |  |
| Ice | 0.02 (0.02) | 0.02 (0.02) |  |
| Other | 0.44 (0.06) | 0.41 (0.06) |  |
| **Tea, coffee, and hot chocolate (cacao)** |  |  |  |
| Tea (in kg) | 1.29 (0.36) | 1.28 (0.32) |  |
| Tea packing (various types) | 0.51 (0.34) | 0.5 (0.34) |  |
| Ground coffee | 1.9 (0.8) | 1.81 (0.73) |  |
| Green seed coffee | 0.13 (0.09) | 0.12 (0.09) |  |
| Coffee substitutes (Nescafe) | 0.06 (0.02) | 0.06 (0.02) |  |
| Cocoa | 0.07 (0.03) | 0.06 (0.03) |  |
| Cappuccino | 0.17 (0.09) | 0.16 (0.09) |  |
| Other | 0.1 (0.03) | 0.1 (0.03) |  |
| **Total spices, salt & other preserves** |  |  |  |
| Black pepper | 0.21 (0.15) | 0.2 (0.14) |  |
| Assorted spices | 0.42 (0.03) | 0.41 (0.03) |  |
| Cardamom | 0.03 (0.01) | 0.03 (0.01) |  |
| Sumac | 0.11 (0.03) | 0.1 (0.03) |  |
| Other spices | 0.36 (0.06) | 0.32 (0.06) |  |
| Sesame | 0.15 (0.02) | 0.14 (0.02) |  |
| Dried sage | 0.12 (0.12) | 0.12 (0.11) |  |
| Treated thyme | 0.49 (0.06) | 0.44 (0.06) |  |
| Salt | 8.93 (0.32) | 8.67 (0.27) |  |
| Lemon salt | 0.07 (0.01) | 0.07 (0) |  |
| Tehinah | 1.1 (0.1) | 0.94 (0.09) |  |
| Green olive | 1.67 (0.79) | 1.62 (0.73) |  |
| Soup with noodle | 0.19 (0.01) | 0.18 (0.01) |  |
| Soup (cubes) | 0.24 (0.03) | 0.22 (0.03) |  |
| Coconut, rasped | 0.22 (0.04) | 0.2 (0.03) |  |
| Vinegar | 0.09 (0) | 0.08 (0) |  |
| Pickles | 0.73 (0.14) | 0.64 (0.12) |  |
| Ketch up | 0.09 (0.05) | 0.08 (0.04) |  |
| Yeast | 0.48 (0.2) | 0.45 (0.19) |  |
| Vanilla | 0.08 (0.03) | 0.07 (0.02) |  |
| Baking powder | 0.07 (0.02) | 0.07 (0.02) |  |
| Potato products (potato chips) | 2.54 (0.09) | 2.2 (0.09) |  |
| Other preserves | 2.38 (0.22) | 2.12 (0.19) |  |
| **Beverages** |  |  |  |
| Mineral water | 1.59 (1.07) | 1.34 (1) |  |
| Juice liquid | 22.34 (5.77) | 20.2 (5.49) |  |
| Juice powder | 0.06 (0.04) | 0.05 (0.03) |  |
| Concentrated juice | 3.76 (0.67) | 3.4 (0.59) |  |
| Soft drinks, can | 3.49 (0.29) | 3.26 (0.26) |  |
| Soft drinks, bottles | 0.43 (0.24) | 0.44 (0.22) |  |
| Soft drinks, family size | 46.05 (13.19) | 42.8 (11.39) |  |
| Natural fruit juice | 0.55 (0.4) | 0.48 (0.37) |  |
