# Supplementary material for: Reducing chronic disease through changes in food aid: A microsimulation of nutrition and cardiometabolic disease among Palestinian refugees in the Middle East
Source: PLoS Med. 2018 Nov 20;15(11):e1002700. doi: 10.1371/journal.pmed.1002700 (PMC6245519; doi:10.1371/journal.pmed.1002700)
Supplement: S2 Table — (DOCX) [file pmed.1002700.s003.docx]

S2 Table: Distribution of content variations by food category ^1,2^.

| Food category | Sodium Mean (SD), mg/kcal | Potassium Mean (SD), mg/kcal | Polyunsaturated Fatty Acids Mean (SD), microg/kcal | Monounsaturated Fatty Acids Mean (SD), microg /kcal | Saturated Fatty Acids Mean (SD), microg/kcal |
| --- | --- | --- | --- | --- | --- |
| Cereals | 1.65 (1.06) | 0.62 (0.41) | 8.82 (6.96) | 9.35 (6.99) | 8.77 (7.86) |
| Tubers, pulses, legumes, and nuts | 1.98 (1.55) | 2.34 (0.75) | 10.72 (7.70) | 11.28 (8.93) | 4.92 (3.62) |
| Fruits and vegetables | 0.06 (0.17) | 3.14 (1.32) | 1.56 (2.74) | 2.20 (9.95) | 1.08 (2.69) |
| Animal products | 4.37 (12.70) | 1.72 (1.19) | 9.86 (7.83) | 19.36 (9.66) | 14.41 (8.24) |
| Additional Oils and fats | 2.08 (2.40) | 0.14 (0.24) | 40.54 (23.20) | 27.92 (11.38) | 28.06 (22.11) |
| Sugars | 0.90 (1.65) | 7.85 (16.95) | 0.81 (2.00) | 2.87 (6.03) | 2.48 (7.37) |

1. Food and Agricultural Organization. International Network of Food Data Systems. Rome: FAO; 2017.

2. US Department of Agriculture. USDA National Nutrient Database for Standard Reference. Beltville: USDA; 2016.
