# Supplementary material for: Reducing chronic disease through changes in food aid: A microsimulation of nutrition and cardiometabolic disease among Palestinian refugees in the Middle East
Source: PLoS Med. 2018 Nov 20;15(11):e1002700. doi: 10.1371/journal.pmed.1002700 (PMC6245519; doi:10.1371/journal.pmed.1002700)
Supplement: S3 Table — (DOCX) [file pmed.1002700.s004.docx]

S3 Table: Alternative results when sampling from the distribution of potential under- and over-reporting by food class ^1^. Compare to main text Table 5. Model-derived estimates of changes in chronic disease outcome measures attributable to a change from food parcel to electronic debit card delivery of food aid; a change from food parcel to cash aid; or a change from food parcel to alternative food parcel with less grain and increased fruit and vegetable content.

| Outcome, per 1000 person-years | Mean change (95% CI) attributable to change in aid | | |
| --- | --- | --- | --- |
| Change from traditional (in kind) food aid to: | **Debit card** | **Cash** | **Alternative parcel** |
| Hypertension incidence | +0.15 (-3.05, +2.60) | +0.05 (-3.36, +2.59) | -0.05 (-0.09, -0.01) |
| Type 2 diabetes incidence | +0.71 (-3.14, +6.96) | +1.22 (-2.94, +8.03) | -0.19 (-0.22, -0.14) |
| Cardiovascular disease events (myocardial infarction or stroke) | +0.36 (-0.97, +1.04) | +0.46 (-0.94, +1.25) | -0.18 (-0.19, -0.17) |
| End-stage renal disease | +0.08 (-0.34, +0.72) | +0.17 (-0.33, +0.84) | -0.04 (-0.24, -0.03) |
| Diabetic neuropathy | +0.18 (-1.17, +2.10) | +0.35 (-1.09, +2.41) | -0.05 (-0.08, -0.03) |
| Diabetic retinopathy | +0.24 (-1.52, +2.67) | +0.51 (-1.45, +3.05) | -0.08 (-0.11, -0.06) |
| All-cause mortality | +0.13 (-0.51, +1.10) | +0.24 (-0.46, +1.30) | -0.02 (-0.04, -0.01) |

1. Marks GC, Hughes MC, van der Pols JC. Relative Validity of Food Intake Estimates Using a Food Frequency Questionnaire Is Associated with Sex, Age, and Other Personal Characteristics. J Nutr [Internet]. 2006 Feb 1 [cited 2018 May 17];136(2):459–65. Available from: https://academic.oup.com/jn/article/136/2/459/4743763
