# Supplementary material for: Reducing chronic disease through changes in food aid: A microsimulation of nutrition and cardiometabolic disease among Palestinian refugees in the Middle East
Source: PLoS Med. 2018 Nov 20;15(11):e1002700. doi: 10.1371/journal.pmed.1002700 (PMC6245519; doi:10.1371/journal.pmed.1002700)
Supplement: S4 Table — (DOCX) [file pmed.1002700.s005.docx]

S4 Table: Impact inventory for elements included and not included in the cost-effectiveness analysis of electronic debit card delivery of food aid (“e-vouchers”), versus traditional food parcel delivery (“in-kind” food aid). The impact inventory is based on the template provided in the 2016 Recommendations from the Second Panel on Cost-effectiveness in Health and Medicine ^1^.

| Sector | Type of impact | Included in analysis from… perspective? | | Sources of evidence  (see Table 2) |
| --- | --- | --- | --- | --- |
|  |  | Healthcare sector | Societal |  |
| Formal healthcare sector | | | | |
| Health | *Health outcomes (effects):* |  |  |  |
|  | Longevity effects | Yes | Yes | United Nations; Globorisk equations; and Risk Equations for Complications of Type 2 Diabetes Mellitus |
|  | Health-related quality-of-life effects | Yes | Yes | Global Burden of Disease Study |
|  | *Medical costs:* |  |  |  |
|  | Paid for by third-party payers | Yes | Yes | United Nations; International Drug Price Indicator Guide |
|  | Paid for by patients out-of-pocket | Yes | Yes | United Nations; International Drug Price Indicator Guide |
|  | Future related medical costs (payers and patients) | Yes | Yes | United Nations; International Drug Price Indicator Guide |
|  | Future unrelated medical costs (payers and patients) | Yes | Yes | United Nations; International Drug Price Indicator Guide |
| Informal healthcare sector | | | | |
| Health | Patient-time costs | NA | No | NA |
|  | Unpaid caregiver-time costs | NA | No | NA |
|  | Transportation costs | NA | No | NA |
| Non-health care sectors | | | | |
| Productivity | Labor market earnings lost | NA | No | NA |
|  | Cost of unpaid lost productivity due to illness | NA | No | NA |
|  | Cost of uncompensated household production | NA | No | NA |
| Consumption | Future consumption unrelated to health | NA | No | NA |
| Social services | Cost of social services as part of intervention | NA | Yes | World Food Program |
| Legal or Criminal Justice | Number of crimes related to intervention | NA | No | NA |
|  | Cost of crimes related to intervention | NA | No | NA |
| Education | Impact of intervention on educational achievement of population | NA | No | NA |
| Housing | Cost of intervention on home improvements | NA | No | NA |
| Environment | Production of toxic waste pollution by intervention | NA | No | NA |

1. Sanders GD, Neumann PJ, Basu A, Brock DW, Feeny D, Krahn M, et al. Recommendations for Conduct, Methodological Practices, and Reporting of Cost-effectiveness Analyses: Second Panel on Cost-Effectiveness in Health and Medicine. JAMA [Internet]. 2016;316(10):1093–103. Available from: http://www.ncbi.nlm.nih.gov/pubmed/27623463
