# Supplementary material for: Reducing chronic disease through changes in food aid: A microsimulation of nutrition and cardiometabolic disease among Palestinian refugees in the Middle East
Source: PLoS Med. 2018 Nov 20;15(11):e1002700. doi: 10.1371/journal.pmed.1002700 (PMC6245519; doi:10.1371/journal.pmed.1002700)
Supplement: S5 Table — (DOCX) [file pmed.1002700.s006.docx]

S5 Table: Demographics of the healthcare input data versus the Palestinian refugee population. The population distribution comparison was used to estimate frequency weights for survey weighting ^1–4^.

| Location | Jordan (2,175,491 registered refugees) | Lebanon (463,664 registered refugees) | Syria (543,014 registered refugees) | West Bank (809,738 registered refugees) | Gaza (1,348,536 registered refugees) |
| --- | --- | --- | --- | --- | --- |
| % Aged <14 |  |  |  |  |  |
| Population | 30 | 24 | 30 | 34 | 40 |
| Input data | 37 | 26 | 33 | 31 | 43 |
|  |  |  |  |  |  |
| % Male |  |  |  |  |  |
| Population | 32 | 47 | 40 | 51 | 38 |
| Input data | 32 | 49 | 40 | 45 | 28 |

1. Chaaban J, Salti N, Ghattas H, Irani A, Ismail T, Batlouni L. Survey on the Socioeconomic Status of Palestine Refugees in Lebanon 2015. 2015; Available from: https://www.unrwa.org/sites/default/files/content/resources/survey_on_the_economic_status_of_palestine_refugees_in_lebanon_2015.pdf

2. United Nations Relief and Works Agency. Palestine Refugees Demographics. 2017;(June):2017.

3. Sabatinelli G. Facing socio-economic decline : Delivering health to Palestine refugees. 2008;

4. United Nations Relief and Works Agency. Palestine refugees in the west bank. 2011;
