# Supplementary material for: Reducing chronic disease through changes in food aid: A microsimulation of nutrition and cardiometabolic disease among Palestinian refugees in the Middle East
Source: PLoS Med. 2018 Nov 20;15(11):e1002700. doi: 10.1371/journal.pmed.1002700 (PMC6245519; doi:10.1371/journal.pmed.1002700)
Supplement: S6 Table — (DOCX) [file pmed.1002700.s007.docx]

S6 Table: Energy metabolism parameter values used in the model for body weight change from a change in caloric intake ^1,2^.

| Parameter | Definition | Value |
| --- | --- | --- |
| *η_f_* | Fat synthesis efficiency, kcal/kg | 230 |
| *η_l_* | Protein synthesis efficiency, kcal/kg | 180 |
| *ρ_f_* | Energy content per unit change in body fat, kcal/kg | 9400 |
| *ρ_l_* | Energy content per unit change in lean tissue, kcal/kg | 1800 |
| *α* | Relative change in lean mass per change in fat mass | 0.5 |
| *β* | Adaptive thermogenesis parameter | 0.2 |
| *γ_f_* | Resting metabolic rate of fat, kcal/kg/d | 3.6 |
| *γ_l_* | Resting metabolic rate of lean tissue, kcal/kg/d | 22.0 |

1. Hall KD, Sacks G, Chandramohan D, Chow CC, Wang YC, Gortmaker SL, et al. Quantification of the effect of energy imbalance on bodyweight. Lancet. 2011;378(9793):826–37.

2. Hall KD, Jordan PN. Modeling weight-loss maintenance to help prevent body weight regain. Am J Clin Nutr. 2008 Dec;88(6):1495–503.
