# Supplementary material for: Reducing chronic disease through changes in food aid: A microsimulation of nutrition and cardiometabolic disease among Palestinian refugees in the Middle East
Source: PLoS Med. 2018 Nov 20;15(11):e1002700. doi: 10.1371/journal.pmed.1002700 (PMC6245519; doi:10.1371/journal.pmed.1002700)
Supplement: S7 Table — (DOCX) [file pmed.1002700.s008.docx]

S7 Table: Globorisk equation coefficients ^1^.

| Risk factor | Main effect coefficient | Age interaction coefficient |
| --- | --- | --- |
| Systolic blood pressure (per 10 mmHg) | 0.3070 | -0.0022 |
| Total cholesterol (per 1 mmol/L) | 0.6149 | -0.0069 |
| Diabetes | 1.4753 | -0.0132 |
| Female with diabetes | 0.4050 | ·· |
| Smoker | 1.8567 | -0.0221 |
| Female smoker | 0.3254 | ·· |

1. Ueda P, Woodward M, Lu Y, Hajifathalian K, Al-Wotayan R, Aguilar-Salinas CA, et al. Laboratory-based and office-based risk scores and charts to predict 10-year risk of cardiovascular disease in 182 countries: a pooled analysis of prospective cohorts and health surveys. Lancet Diabetes Endocrinol. 2017 Mar;5(3):196–213.
