# Supplementary material for: Reducing chronic disease through changes in food aid: A microsimulation of nutrition and cardiometabolic disease among Palestinian refugees in the Middle East
Source: PLoS Med. 2018 Nov 20;15(11):e1002700. doi: 10.1371/journal.pmed.1002700 (PMC6245519; doi:10.1371/journal.pmed.1002700)
Supplement: S8 Table — (DOCX) [file pmed.1002700.s009.docx]

S8 Table: RECODe equation coefficients ^1^.

| Parameter | End-stage renal disease coefficient | Diabetic neuropathy coefficient | Diabetic retinopathy coefficient | All-cause mortality coefficient |
| --- | --- | --- | --- | --- |
| Hazard (*λ*) | 0.96 | 0.57 | 0.59 | 0.95 |
| Age,years | -0.01938 | 0.02285 | 0.03022 | 0.06703 |
| Women | -0.01129 | 0.22640 | -0.18680 | -0.15290 |
| Black | 0.08812 | -0.16770 | -0.09448 | -0.02393 |
| Hispanic or Latino | 0.23380 | .. | .. | .. |
| Tobacco smoking, current | 0.14830 | .. | .. | 0.53990 |
| Systolic blood pressure, mmHg | 0.00303 | 0.00824 | 0.00456 | -0.00299 |
| Cardiovascular disease history | -0.02164 | 0.11270 | 0.26672 | 0.58880 |
| Blood pressure- lowering drugs | -0.07952 | 0.06393 | 0.18192 | 0.08776 |
| Oral diabetes drugs | -0.12560 | -0.23490 | -0.25747 | .. |
| Statin drugs | .. | .. | .. | -0.26810 |
| Anticoagulants | 0.03199 | .. |  | 0.40360 |
| HbA1c, % | 0.13690 | 0.14490 | 0.18866 | 0.16590 |
| Total cholesterol, mg/dL (mmol/L) | -0.00111 (-0.04327) | -0.00017 (-0.00663) | 0.00219 (0.08536) | -0.00095 (-0.03703) |
| HDL cholesterol, mg/dL (mmol/L) | 0.00629 (0.23902) | 0.00545 (0.20710) | -0.00539 (-0.20482) | -0.00438 (-0.16644) |
| Serum creatinine, mg/dL (micromol/L) | 0.86090 (0.00986) | 0.69470 (0.00795) | 0.60442 (0.00692) | 0.35970 (0.00412) |
| Urine albumin:creatinine ratio, mg/g (mg/mmol) | 0.00036 (0.00316) | 0.00020 (0.00176) | .. | 0.00039 (0.00342) |

1. Basu S, Sussman JB, Berkowitz SA, Hayward RA, Yudkin JS. Development and validation of Risk Equations for Complications Of type 2 Diabetes (RECODe) using individual participant data from randomised trials. Lancet Diabetes Endocrinol [Internet]. 2017 Oct 1 [cited 2018 Mar 8];5(10):788–98. Available from: https://www.sciencedirect.com/science/article/pii/S2213858717302218
