# Supplementary material for: Reducing chronic disease through changes in food aid: A microsimulation of nutrition and cardiometabolic disease among Palestinian refugees in the Middle East
Source: PLoS Med. 2018 Nov 20;15(11):e1002700. doi: 10.1371/journal.pmed.1002700 (PMC6245519; doi:10.1371/journal.pmed.1002700)

S1 Figure: Covariance between key demographic, dietary, and health features. Legend: sbp = systolic blood pressure (mmHg); totchol = total cholesterol (mmol/L); hdlchol = HDL cholesterol (mmol/L); a1c = hemoglobin A1c (%); sercreat = serum creatinine micromol/L); bmi = body mass index (kg/m^2^). The [1:1000] notation indicates this as a random 1,000-person sample for display purposes. Note that self-reported age tends to be reported as exactly 100 for some older adults due to a social means of responding to surveys among older adults, not due to exact age; note this does not affect our results since our analysis was restricted to the 20-79 year old subpopulation. The distribution reflects both people without diabetes and a subset with effective treatment, though there is another subset producing a long right tail.


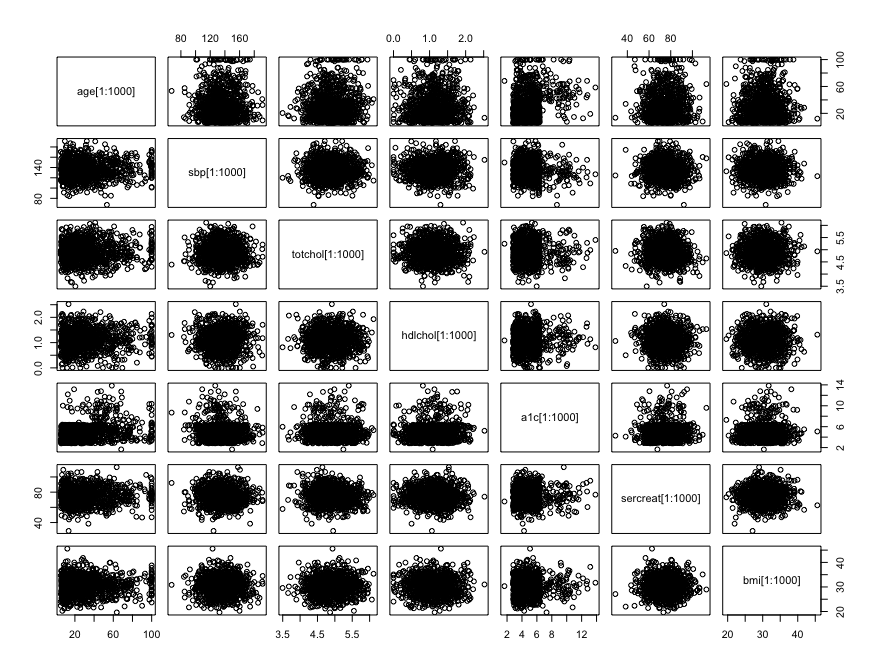

Supplement: S1 Fig — (DOCX) [file pmed.1002700.s011.docx]
